# MambaSeed: A lightweight Mamba-based hybrid vision network for tomato leaf disease recognition

**DOI:** 10.1371/journal.pone.0359350

**Published:** 2026-10-15

**Authors:** XueShen Wang, Heng Zhang, Yayong Chen, YunLong Li, YuJie Qin, ZhongYu Lv, XiangJuan Liu

**Affiliations:** 1 College of Computer and Control Engineering, Qiqihar University, Qiqihar, Heilongjiang, China; 2 State Key Laboratory of Water Engineering Ecology and Environment in Arid Area, Xi’an University of Technology, Xi’an, China; 3 School of Water Resources and Hydro-electric Engineering of XUT, Xi’an University of Technology, Xi’an, China; 4 Heilongjiang Key Laboratory of Big Data Network Security Detection and Analysis, Qiqihar University, Qiqihar, Heilongjiang, China; Instituto Politecnico Nacional, MEXICO

## Abstract

Crop diseases threaten agricultural productivity, creating a need for recognition models that combine accuracy, robustness, and computational efficiency. Existing CNN-, Transformer-, and state-space-based approaches can weaken fine-grained lesion edges and textures when emphasizing global context. We therefore propose MambaSeed, a lightweight hybrid network built on MambaVisionMixer and equipped with the Local Continuous Branch Injection mechanism, hereafter LCBI. This mechanism repeatedly introduces local feature compensation during hierarchical modeling, preserving high-frequency cues while maintaining global dependency modeling. On the Tomato, Corn, and Potato datasets, MambaSeed achieved accuracies of 98.97%, 98.06%, and 95.87%, respectively, with 1.13M parameters and 0.240G MACs. In the batch-one FP32 GPU benchmark, its latency was 3.39 ms/image on an NVIDIA RTX 4090. Without a hardware accelerator, the median CPU latency was 25.35 ms/image with one thread and 15.61 ms/image with four threads. Ablation, cross-architecture frequency analysis, and controlled perturbation tests further showed that LCBI strengthens high-frequency feature retention and maintains stable recognition under visual degradation. Together, these results show that MambaSeed provides a compact balance of accuracy, efficiency, and robustness across both accelerator-based and CPU-only inference settings.

## Introduction

Tomato is an important economic crop and vegetable commodity, and its yield is closely related to food security and farmers’ income. However, tomato cultivation faces serious threats from many diseases, such as Bacterial Spot, Early Blight, and Late Blight. The losses caused by these diseases seriously threaten global tomato supply and farmers’ livelihoods [1]. Therefore, crop disease diagnosis is of great importance. Traditional diagnosis, however, depends on the visual inspection and experience-based judgment of agricultural experts [2]. Such diagnosis is highly subjective and inefficient and cannot fully meet the needs of modern agriculture [3]. With the development of computer vision and machine learning, deep-learning-based crop disease recognition has become an important research direction and has shown clear advantages in both recognition accuracy and automation [4].

In resource-constrained farmland environments, reducing computational complexity while maintaining recognition accuracy has become an urgent problem. CNN- and Transformer-based models have become mainstream baselines for agricultural disease recognition and have given rise to various improved variants targeting specific limitations. For example, some studies introduced a two-stage transfer learning technique combined with partial convolution modules to reduce data dependency and enhance model stability [5]; used a multi-component approach to improve CNN-based citrus disease recognition [6]; adopted a lightweight coordinate attention mechanism together with local binary patterns to effectively improve model performance and robustness [7]; and employed deep channel shuffling to improve feature extraction [8]. These methods can improve recognition performance under complex conditions. Nevertheless, CNNs are constrained by local receptive fields, which limits their ability to model long-range dependencies and makes it difficult to capture sufficient global context in unstructured field scenes. Moreover, illumination changes, image blur, and leaf occlusion in real agricultural settings further degrade robustness. Transformer-based models alleviate some of these issues through self-attention-based global dependency modeling [9]. Some studies have further enhanced the modeling of key regions by introducing improved attention mechanisms or incorporating convolutional structures [10,11]. However, Transformer-based methods usually involve high computational complexity and large parameter counts, which restrict deployment in resource-limited farmland scenarios.

To address the tension between global modeling capability and deployment efficiency, there is a need for a visual model that can capture long-range dependencies while remaining computationally efficient. In recent years, state-space models have attracted growing attention in vision. Representative architectures such as Mamba achieve efficient long-sequence modeling with linear complexity and strike a good balance between performance and efficiency [12]. For example, selective state-space models have been introduced for super-resolution reconstruction of low-resolution agricultural UAV imagery [13,14]. Improved high-order two-dimensional selective scanning has been used to enhance local feature learning at each interaction stage [15]. Other work has combined ideas from quantum-state modeling and graph-convolution-based feature prioritization to build networks that more effectively optimize visual feature extraction and establish multi-scale feature dependencies in complex environments [16]. However, current state-space models in vision still face certain challenges. Although Mamba-like architectures can achieve efficient long-range dependency modeling through selective state-space mechanisms, their modeling process primarily focuses on sequence propagation and exhibits an implicit bias toward low-frequency components over high-frequency ones. In practice, this can lead to insufficient exploitation of frequency-domain information, especially high-frequency information, and thus to the gradual attenuation of fine-grained texture cues during feature transmission [17,18]. In crop disease recognition, lesion regions usually exhibit complex textures and edge structures, which essentially correspond to high-frequency image information. Existing studies have shown that texture features can effectively reflect structural differences in diseased leaf regions. Likewise, explicitly characterizing spatial-frequency distributions through methods such as Gabor filtering and wavelet transforms can further improve the representation of fine-grained lesion information [19,20]. Recent disease-imaging research has also incorporated frequency- and gradient-domain constraints to strengthen the representation of high-frequency pathological details and fine-grained lesion cues [21]. Current visual state-space approaches mainly improve overall performance through modified scanning strategies or structural combinations, rather than explicitly compensating for high-frequency information loss. Motivated by this gap, this study proposes a lightweight hybrid vision network, MambaSeed, built on the mechanism termed Local Continuous Branch Injection, hereafter referred to as LCBI.

With this motivation in place, we first review the existing architectural directions that shape the problem, and then explain how MambaSeed combines state-space modeling with continuous local compensation. The subsequent experiments examine this design from complementary perspectives, covering recognition performance, efficiency, learned representations, ablation behavior, frequency retention, and robustness. The main contributions are as follows:

1. We propose the Local Continuous Branch Injection mechanism. This mechanism introduces a local information compensation pathway during multi-layer feature propagation, thereby alleviating the weakening of fine-grained local information in visual state-space models under sequential modeling.
2. Based on MambaVisionMixer [22], we construct a lightweight hybrid vision network named MambaSeed, which enables collaborative optimization of global modeling and local detail representation while maintaining low parameter count and computational cost.
3. Evaluation across classification performance, model complexity, ablation behavior, frequency characteristics, and controlled perturbations shows that MambaSeed balances accuracy, efficiency, high-frequency information retention, and robustness under the evaluated conditions.

## Related work

This section reviews research on crop disease recognition based on convolutional neural networks, Transformers, and state-space models, and analyzes the advantages and limitations of these three paradigms in terms of local feature extraction, global dependency modeling, and lightweight design.

### CNN-based methods

Convolutional neural networks have long been a mainstream approach for crop disease recognition. By extracting image features layer by layer through convolution, CNNs exhibit a strong capability for modeling textures and edges. Several studies have attempted to improve CNN-based disease recognition through different strategies, including optimizing Faster R-CNN in detection frameworks to improve lesion localization accuracy [23], combining lightweight backbones with attention mechanisms to improve efficiency [24], enhancing representation capability for complex data through 3D convolutions and multi-branch feature fusion [25,26], and incorporating attention mechanisms into residual feature learning to focus on salient discriminative patterns in fine-grained classification [27]. Depthwise-separable convolution has also been explored in lightweight temporal models to improve feature extraction efficiency [28].

Despite these architectural refinements, convolutional modeling still relies primarily on local receptive fields and therefore remains limited in capturing long-range dependencies. In complex farmland environments, this limitation makes it difficult for the model to exploit sufficient contextual information, which in turn weakens the overall quality of feature representation.

### Transformer-based methods

To achieve stronger global modeling in realistic farmland environments, recent studies have explored Transformers and their variants in agricultural vision tasks. Some work has built robust frameworks based on pure vision Transformers for high-resolution weed classification or tomato leaf disease recognition [29,30]. To balance computational efficiency and feature extraction accuracy, other studies have combined lightweight convolutional networks with Transformers to achieve more efficient feature fusion [31]. In agricultural remote sensing, CNN–Transformer hybrid architectures have further integrated convolutional local-detail extraction with Transformer-based long-range modeling to improve the representation of complex spatial structures and boundaries [32]. Transformer-based approaches have also been extended to disease severity estimation and accurate localization in complex scenes through multi-task hybrid architectures combining DETR and Swin Transformer, or through real-time detectors based on RT-DETR [33,34].

From both the algorithmic and deployment perspectives, Transformer models have two key limitations: the computational complexity of global self-attention grows quadratically with image resolution, and Transformers lack the local inductive bias naturally provided by convolutions. As a result, they may be less sensitive to fine-grained local lesion patterns. Under complex and resource-constrained edge conditions in farmland, these limitations increase deployment difficulty and make it challenging to achieve an ideal balance between high-precision local feature capture and lightweight real-time inference.

### State-space-model-based methods

In recent years, state-space models have received increasing attention in vision because of their efficient long-range dependency modeling ability. Representative methods such as Mamba use selective state updates to achieve linear-complexity sequence modeling and have shown a favorable balance between efficiency and performance across multiple vision tasks. In biometric recognition, a Global–Local Vision Mamba integrates parallel CNN and multi-head Mamba branches through a feature interaction unit and further employs neural architecture search to optimize the hybrid architecture [35]. In detection, Mamba modules have been embedded into YOLO frameworks to strengthen overall perception capability [36]. In pest recognition, graph convolution has been fused with Mamba to enhance joint modeling of local structures and global dependencies [37]. In image segmentation, visual Mamba has been incorporated into U-shaped networks to improve segmentation accuracy [38].

Despite these advances, existing hybrid designs mainly enhance local–global representation through architectural fusion or feature interaction, while the layer-wise attenuation of fine-grained high-frequency cues during hierarchical state-space propagation remains less explicitly addressed. In particular, high-frequency texture information tends to attenuate during multi-layer transmission, which weakens the model’s ability to discriminate detailed structures such as lesions. Enhancing local detail representation while maintaining efficient modeling remains an open issue.

### Research motivation and problem definition

The above analysis shows that different methodological paradigms offer complementary strengths for crop disease recognition. CNN-based methods are effective at extracting local textures and edges, but their long-range modeling capability remains limited when broader contextual information is needed. Transformers can model global contextual information, but their computational cost is high and thus less suitable for resource-constrained settings. Recent state-space models provide a better compromise between efficiency and global modeling, yet there is still room to improve their representation of fine-grained local features, especially because high-frequency cues such as lesion textures tend to decay during multi-layer feature propagation. This motivates our central question of how to limit the attenuation of lesion texture and edge information while retaining lightweight global modeling.

## Materials and methods

### MambaSeed: A lightweight hybrid mamba architecture for agricultural disease recognition

In disease recognition, fine-grained cues such as lesion edges and textures are crucial, yet such information tends to weaken as features propagate into deeper layers. Although state-space models are effective at modeling long-range dependencies, their sequential modeling paradigm is limited in preserving local spatial structures, a limitation that becomes even more pronounced in lightweight networks.

To address this problem, this study introduces LCBI. LCBI is a cross-layer information compensation strategy applied along the feature propagation pathway. By continuously injecting local responses during feature evolution, it preserves the stability of the original spatial structure in deep representations and alleviates the layer-wise attenuation of fine-grained information from the perspective of information flow. Based on this strategy, we build a lightweight hybrid vision architecture, MambaSeed, whose overall structure is illustrated in Fig 1. The network adopts a stage-wise design with five stages.

**Fig 1:**
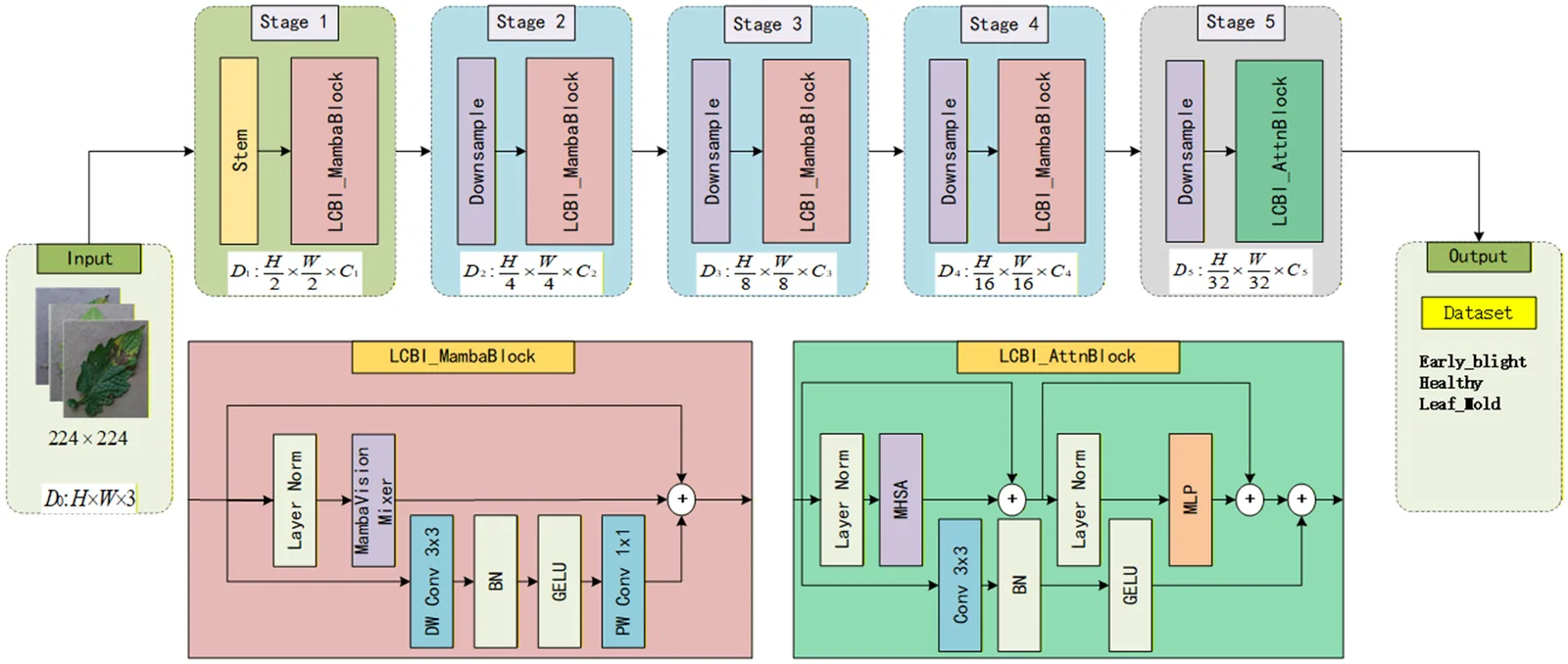
Architecture of MambaSeed.

The input image $I\in {\mathbb{R}}^{H\times W\times 3}$ first passes through a compact Stem module that converts the RGB image into an initial feature representation while performing the first spatial reduction. The Stem is implemented with two consecutive 3×3 convolutional layers, each followed by batch normalization and GELU activation. The first convolution uses stride 2 and padding 1 to reduce the feature resolution, whereas the second uses stride 1 and padding 1 to refine the embedded local patterns without further downsampling. As a result, the Stem outputs feature maps at a resolution of H/2×W/2.

Stage 1 receives the Stem output directly, whereas a dedicated Downsampling layer is inserted before each of Stages 2–5. As implemented in the source code, this layer consists of a 3×3 convolution with stride 2, padding 1, and no bias, followed by batch normalization and GELU activation. The convolution halves the spatial dimensions and projects the channels from the preceding stage to the width required by the next stage. Consequently, Stages 1–5 process feature maps at resolutions of H/2×W/2, H/4×W/4, H/8×W/8, H/16×W/16, and H/32×W/32, respectively.

In each stage, feature propagation is driven by global and local responses. The global response, produced by state-space modeling, captures long-range dependencies across spatial regions, whereas the local convolutional response preserves neighborhood structural cues, including lesion boundaries, texture variations, and subtle patterns. Rather than replacing the global pathway, the local response serves as compensatory information and is continuously injected to reduce fine-grained attenuation in deeper representations.

This design is important for plant disease recognition because discriminative regions are often small, irregular, and texture-dependent. As the hierarchy deepens, feature maps become semantically richer and their spatial structure weakens. Therefore, LCBI introduces local compensation across network levels, keeping local details involved while preserving the efficiency and continuity of the main modeling path.

At the final stage, the feature representation becomes semantically richer but spatially coarser. We therefore employ an MHSA-based LCBI-AttnBlock to balance high-level semantic modeling with local structural preservation. The MHSA pathway captures long-range semantic dependencies, while the parallel local pathway retains disease-related textures and structural cues. Their collaboration improves the representation of visually similar disease categories.

In LCBI, the term continuous does not refer to recursive operations within a single block. Instead, it describes the repeated deployment of the local compensation pathway across multiple network levels. Through this design, local information is not introduced only once or used as an isolated auxiliary branch, but participates in feature propagation throughout the hierarchical network. As a result, fine-grained cues such as lesion edges, texture changes, and small disease spots can be retained and progressively propagated in deeper layers. A detailed computational procedure of MambaSeed is provided in Algorithm S1 of the Supporting Information.

### LCBI-MambaBlock: Global–local collaborative modeling driven by continuous information injection

LCBI-MambaBlock is the block-level implementation of the proposed LCBI mechanism. Rather than treating the block as a direct summation of several feature maps, we formulate its computation as a residual response injection process. Given a two-dimensional feature map $X\in {\mathbb{R}}^{B\times C\times H\times W}$, the residual injection operator RI(·) fuses one or more response terms with the base representation through element-wise residual addition. The operators Π(·) and $\Pi^{-1}(\cdot )$ denote feature-map-to-sequence rearrangement and its inverse operation, respectively.

The sequence representation used by the global pathway is defined as


$$\begin{array}{c} T=\Pi (X),\;T\in {\mathbb{R}}^{B\times HW\times C}. \end{array}$$ (1)

The global response is produced by the MambaVisionMixer ${ℳ}_{\theta }$ after LayerNorm. It is then modulated by the layer-scale parameter γ, regularized by DropPath ${𝒟}_{p}$, and restored to the original spatial layout:


$$\begin{array}{c} R_{global}^{m}(X)=\Pi^{-1}\left({𝒟}_{p}\left(\gamma ⊙{ℳ}_{\theta }(LN(T))\right)\right). \end{array}$$ (2)

In the actual mixer, ${ℳ}_{\theta }$ performs selective state-space modeling. The input sequence is linearly projected and split into two paths; one path generates the dynamic state-space parameters and is processed by selective scan, while the other path provides a parallel gated response. This design enables long-range dependency modeling with linear-complexity sequence propagation. Therefore, $R_{global}^{m}(X)$ mainly carries contextual and semantic dependency information.

For local feature modeling, *X* remains in its original two-dimensional spatial layout. The local pathway adopts a depthwise-separable convolution comprising a 3×3 depthwise convolution, batch normalization, GELU activation, and a 1×1 pointwise convolution. The depthwise convolution captures neighborhood-level textures and lesion boundaries, while the pointwise convolution, denoted as PW Conv in Fig 1, mixes information across channels after spatial filtering:


$$\begin{array}{c} R_{local}^{m}(X)={Conv}_{1\times 1}\left(GELU\left(BN\left({DWConv}_{3\times 3}(X)\right)\right)\right). \end{array}$$ (3)

The depthwise–pointwise decomposition keeps the repeatedly deployed local pathway computationally lightweight. Similar depthwise-separable designs have been shown to reduce computational complexity and parameter count while retaining discriminative high-level representations [39]. In MambaSeed, this efficiency is particularly important because the local branch is repeatedly introduced across hierarchical stages, allowing local structural compensation without substantially increasing the overall model cost. The final LCBI-MambaBlock update is formulated as:


$$\begin{array}{c} Y_{m}=RI\left(X;\left\{R_{global}^{m}(X),R_{local}^{m}(X)\right\}\right). \end{array}$$ (4)

In this formulation, the original feature *X* is treated as the residual carrier, the Mamba response provides global semantic propagation, and the local convolutional response is continuously injected as structural compensation. Thus, LCBI-MambaBlock preserves the efficient global modeling ability of MambaVisionMixer while maintaining the local disease-related patterns required for fine-grained recognition.

### LCBI-AttnBlock: Maintaining local consistency during semantic modeling

In the higher layers of the network, feature representations are dominated by global semantics, while internal spatial structural information becomes relatively weak. To prevent local information from being further weakened during semantic modeling, this study introduces LCBI-AttnBlock and extends the residual injection idea to the high-level attention stage. The input feature is first rearranged into a sequence:


$$\begin{array}{c} T_{0}=\Pi (X). \end{array}$$ (5)

Global semantic modeling is then performed by multi-head self-attention through the residual injection operator:


$$\begin{array}{c} T_{1}=RI\left(T_{0};{𝒟}_{p}\left(MHSA\left(LN(T_{0})\right)\right)\right). \end{array}$$ (6)

The channel representation is further updated by the feed-forward network:


$$\begin{array}{c} T_{2}=RI\left(T_{1};{𝒟}_{p}\left(MLP\left(LN(T_{1})\right)\right)\right). \end{array}$$ (7)

During high-level semantic modeling, the local response is extracted directly from the original two-dimensional input feature, rather than from the attention-updated sequence:


$$\begin{array}{c} R_{local}^{a}(X)=GELU\left(BN\left({Conv}_{3\times 3}(X)\right)\right). \end{array}$$ (8)

Unlike LCBI-MambaBlock, LCBI-AttnBlock employs a standard 3×3 convolution in its local pathway. The Mamba-based block is repeatedly used throughout the hierarchical backbone and therefore adopts a depthwise-separable convolution to control the accumulated computational cost. In contrast, LCBI-AttnBlock appears only once in the final stage, where the feature resolution has been reduced to H/32×W/32. At this resolution, a standard convolution introduces limited additional overhead while jointly modeling spatial neighborhoods and cross-channel interactions. It therefore provides channel-coupled local compensation that complements the global representation produced by MHSA.

The final output of LCBI-AttnBlock is obtained by injecting the local structural response into the restored semantic feature:


$$\begin{array}{c} Y_{a}=RI\left(\Pi^{-1}(T_{2});R_{local}^{a}(X)\right). \end{array}$$ (9)

Because the local response is computed directly from *X*, it retains spatial details before the attention and feed-forward transformations. Injecting this response after restoring *T*_2_ to the feature-map layout therefore preserves local textures while maintaining the high-level semantic representation.

## Experimental setup and results analysis

### Training settings and datasets

Training, classification, and GPU efficiency measurements were conducted on an Intel Xeon Gold 6430 and NVIDIA GeForce RTX 4090 server using Python 3.10.13, PyTorch 2.6.0 + cu124, and CUDA 12.4; full environment details are provided in Supplementary Table S8 in S1 Text. The PlantVillage dataset was used, comprising Tomato with 10,000 images from 10 classes, Corn with 3,852 images from 4 classes, and Potato with 9,778 images from 9 classes [40], as shown in Figs 2–4.

**Fig 2:**
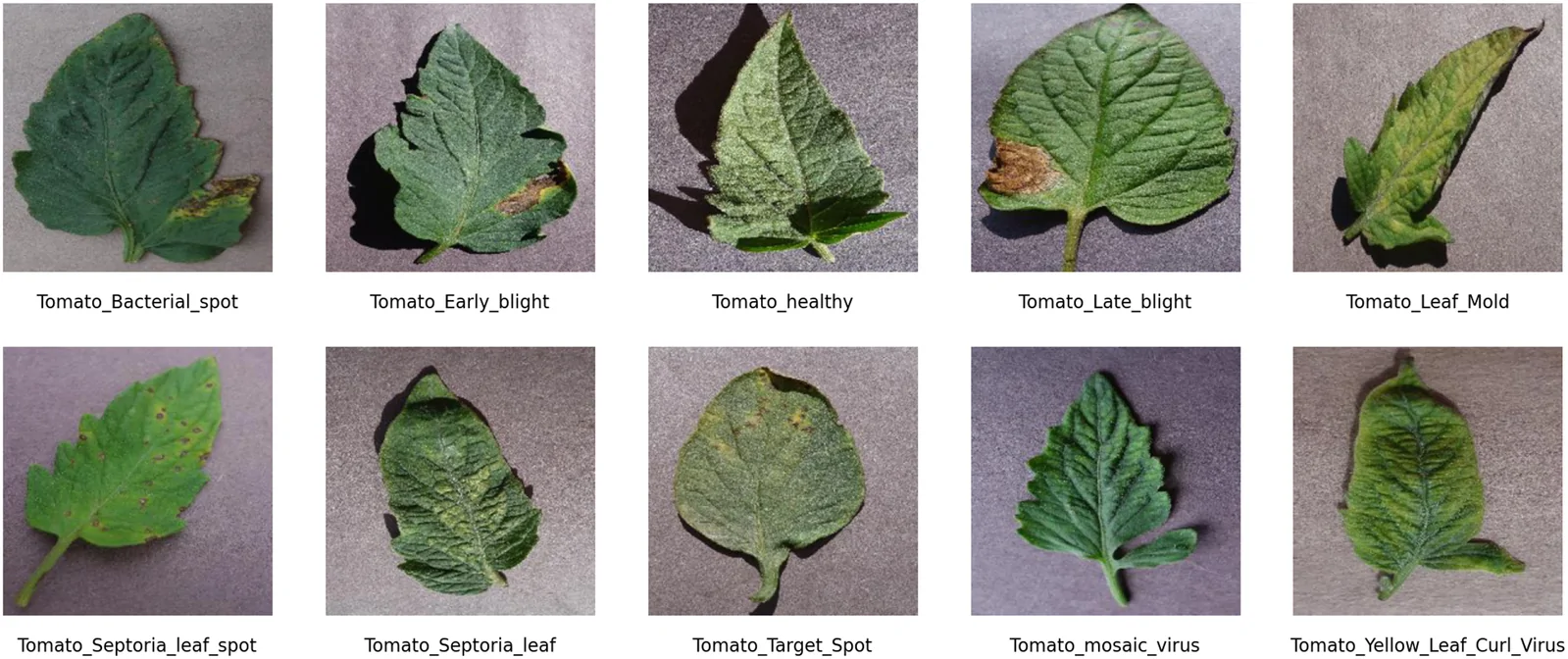
Representative examples of the tomato leaf dataset.

**Fig 3:**
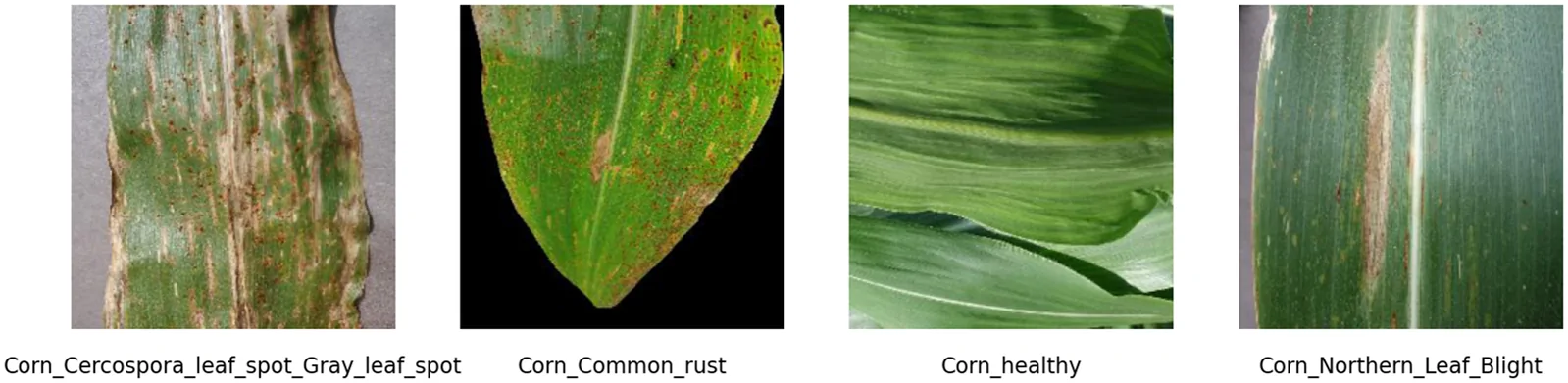
Representative examples of the corn leaf dataset.

**Fig 4:**
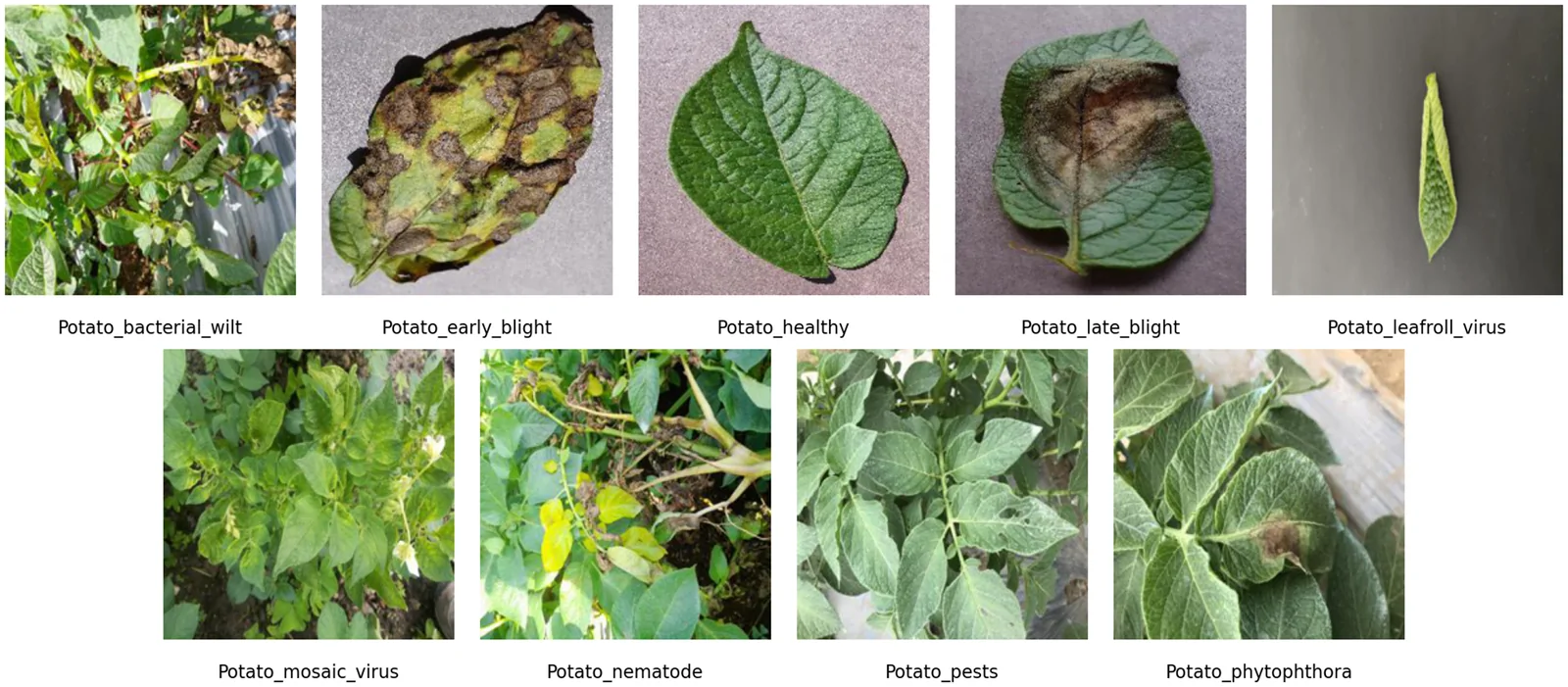
Representative examples of the potato leaf dataset.

The class-balanced Tomato subset supported the primary comparison, while Corn and Potato retained their original class compositions for complementary evaluation. Within each class, a fixed seed of 42 was used for the 60:20:20 split, and identical partitions were retained across models; detailed counts are provided in Supplementary Tables S1–S3 in S1 Text. For visual context, one image per class from each of the Tomato, Corn, and Potato datasets was manually selected independently of model predictions and experimental outcomes.

Because comparisons between learning architectures can be influenced by preprocessing and evaluation choices as well as by the models themselves [41], all models were trained and evaluated on the fixed partitions described above. Inputs were resized to 224×224 and normalized with ImageNet statistics, with random horizontal flipping, rotation within $\pm 15^{\circ }$, and mild color jitter applied only during training. Checkpoints were selected on the validation set, leaving the test set for final reporting. The core hyperparameters used to train MambaSeed are summarized in Table 1. Their selection rationale, together with the exact benchmark variants and complete training protocol, is provided in Supplementary Tables S4 and S5 in S1 Text.

**Table 1:** Core hyperparameters.

Hyperparameter	Value
Input resolution	224×224
Epochs	50
Batch size	64
Optimizer	AdamW
Learning rate	0.0004
Loss function	CrossEntropyLoss
Expansion factor	3

### Feature distribution analysis

Retaining discriminative information while reducing redundant components is important for effective feature representation [42]. To examine whether the classification results were reflected in the learned feature space, we compared the penultimate embeddings of MambaSeed with those of MambaVision-T [22], MobileMamba-S6 [43], ViT-Tiny [44], MobileNetV3 [45], MobileViTv3 [46], ShuffleNetV2 [47], EMOv2-1M [48], and InceptionMamba [49]. The same 2,000 Tomato test images were used for every model, and differences in embedding dimension were removed through model-wise standardization and PCA reduction to 50 dimensions. The resulting features were projected with one shared t-SNE configuration using perplexity 50, PCA initialization, 3,000 iterations, and random state 42, without model-specific tuning or projection selection; complete settings are reported in Supplementary Table S7 in S1 Text. As shown in Fig 5, MambaSeed forms comparatively compact class clusters with clearer separation and less overlap, providing a feature-space view that is consistent with its classification results.

**Fig 5:**
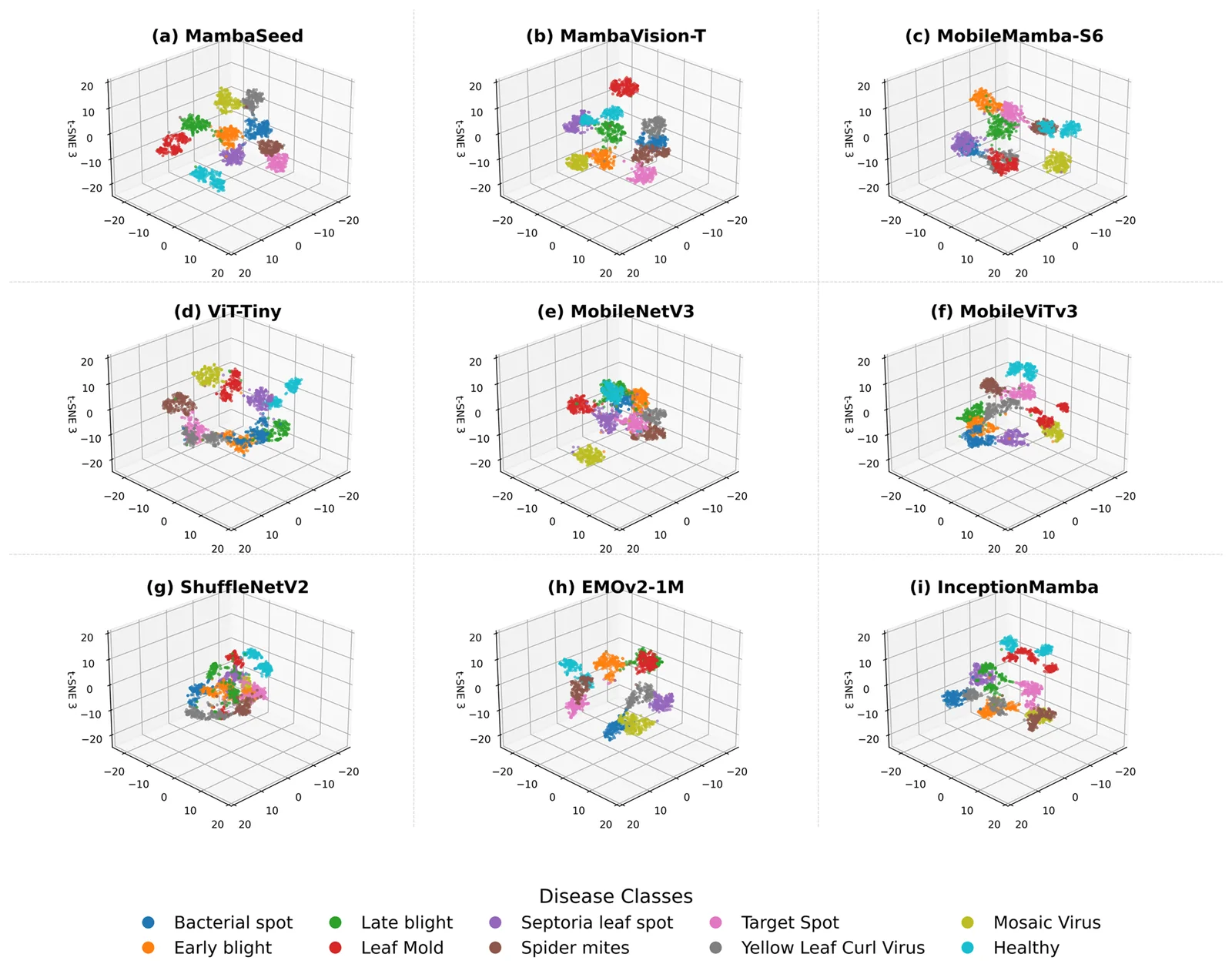
Three-dimensional t-SNE feature visualization of comparative models.

### Experimental results and analysis

To comprehensively evaluate the effectiveness of MambaSeed for plant disease recognition, we compared the proposed model with a set of representative lightweight baselines across classification accuracy, model complexity, inference efficiency, and robustness.

#### Accuracy comparison across datasets.

Fig 6 compares the same model set on the Tomato, Corn, and Potato datasets. MambaSeed achieved accuracies of 98.97%, 98.06%, and 95.87%, respectively, showing consistent recognition across the class-balanced Tomato subset and the complementary crop datasets. These results extend the primary comparison beyond a single benchmark without relying on greater model scale. Confusion matrices on the Tomato dataset are provided in Supplementary Fig. S1 in S1 Text.

**Fig 6:**
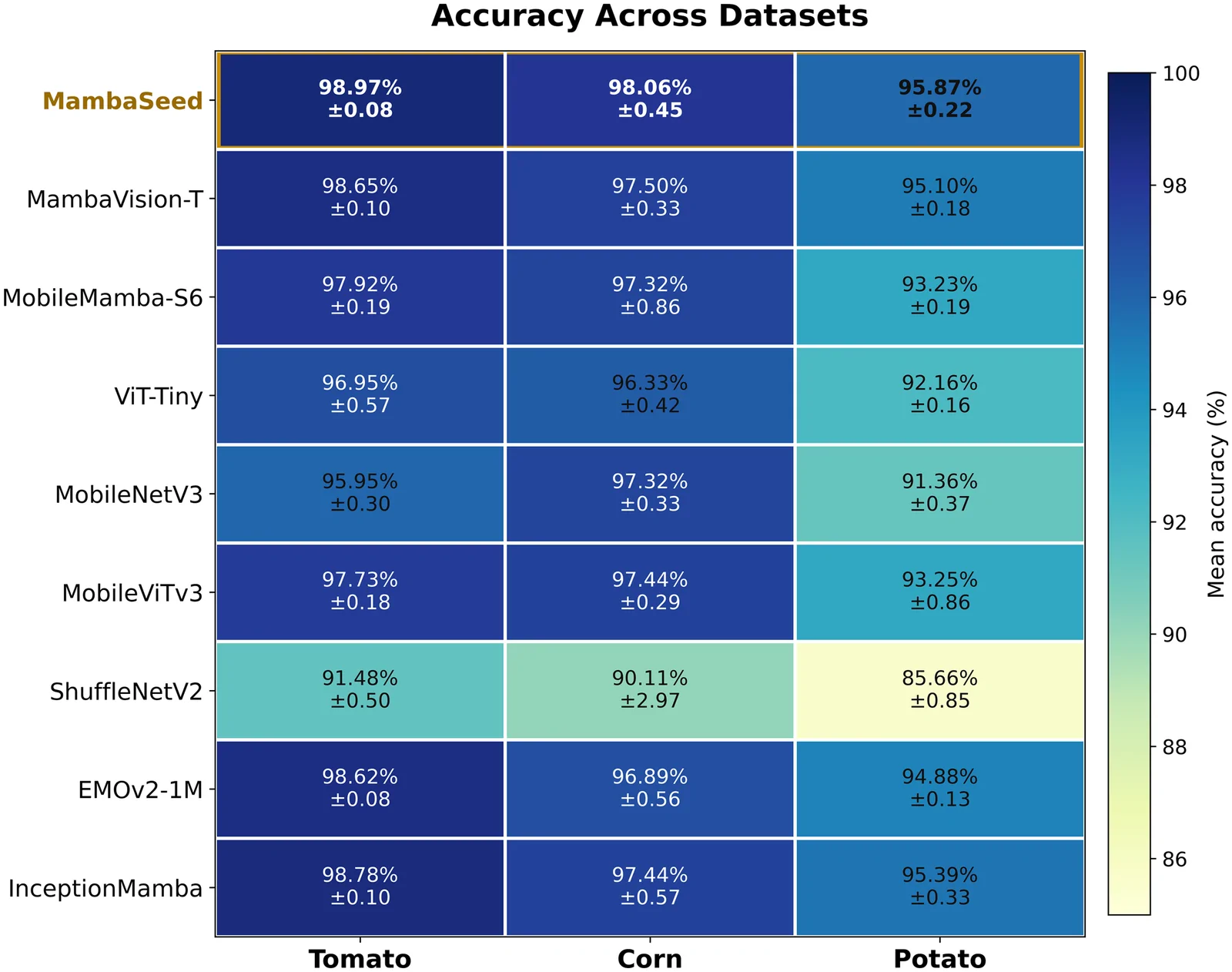
Accuracy comparison across the Tomato, Corn, and Potato datasets.

#### Model complexity and inference efficiency.

All models were profiled at batch size 1 using a common 3×224×224 FP32 input and 10-class output heads. As shown in Fig 7, MambaSeed required 1.13M parameters and 0.240G MACs while reaching a GPU latency of 3.39 ms/image. Although individual compact baselines were smaller or faster, MambaSeed coupled strong cross-dataset accuracy with modest computational demand, whereas the larger Mamba- and hybrid-based models incurred higher costs. Full profiling details are provided in Supplementary Tables S8–S11 in S1 Text.

**Fig 7:**
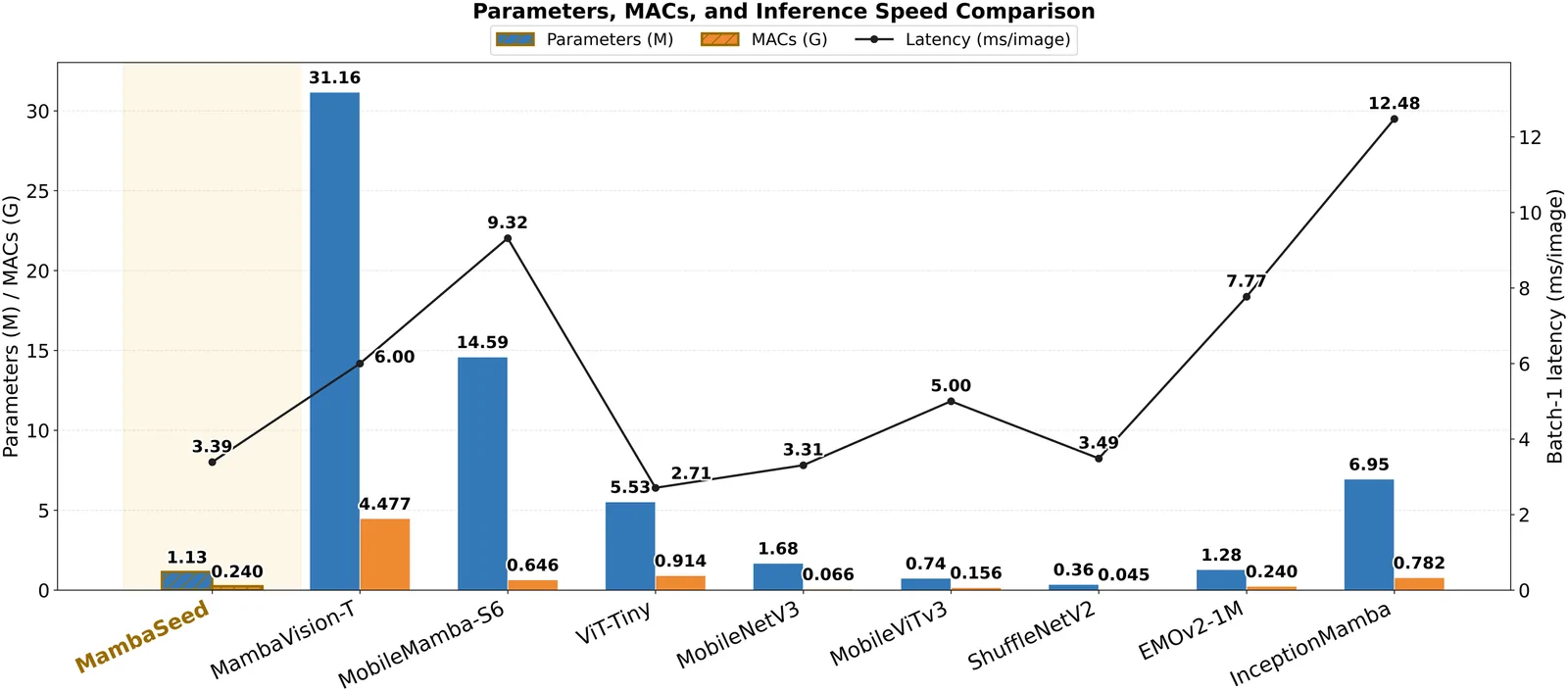
Comparison of model complexity and inference efficiency.

We further repeated the comparison without hardware acceleration on an Intel Xeon Gold 6430 CPU. As shown in Fig 8, MambaSeed reached median latencies of 25.35 and 15.61 ms/image under one- and four-thread settings, respectively, remaining within the 33.3-ms forward-pass budget associated with a 30-FPS stream. Although MobileNetV3 and ShuffleNetV2 ran faster, MambaSeed retained a better balance between inference efficiency and recognition performance, while the larger MambaVision-T, MobileMamba-S6, and InceptionMamba models were substantially slower. The complete CPU protocol is reported in Supplementary Table S12 in S1 Text.

**Fig 8:**
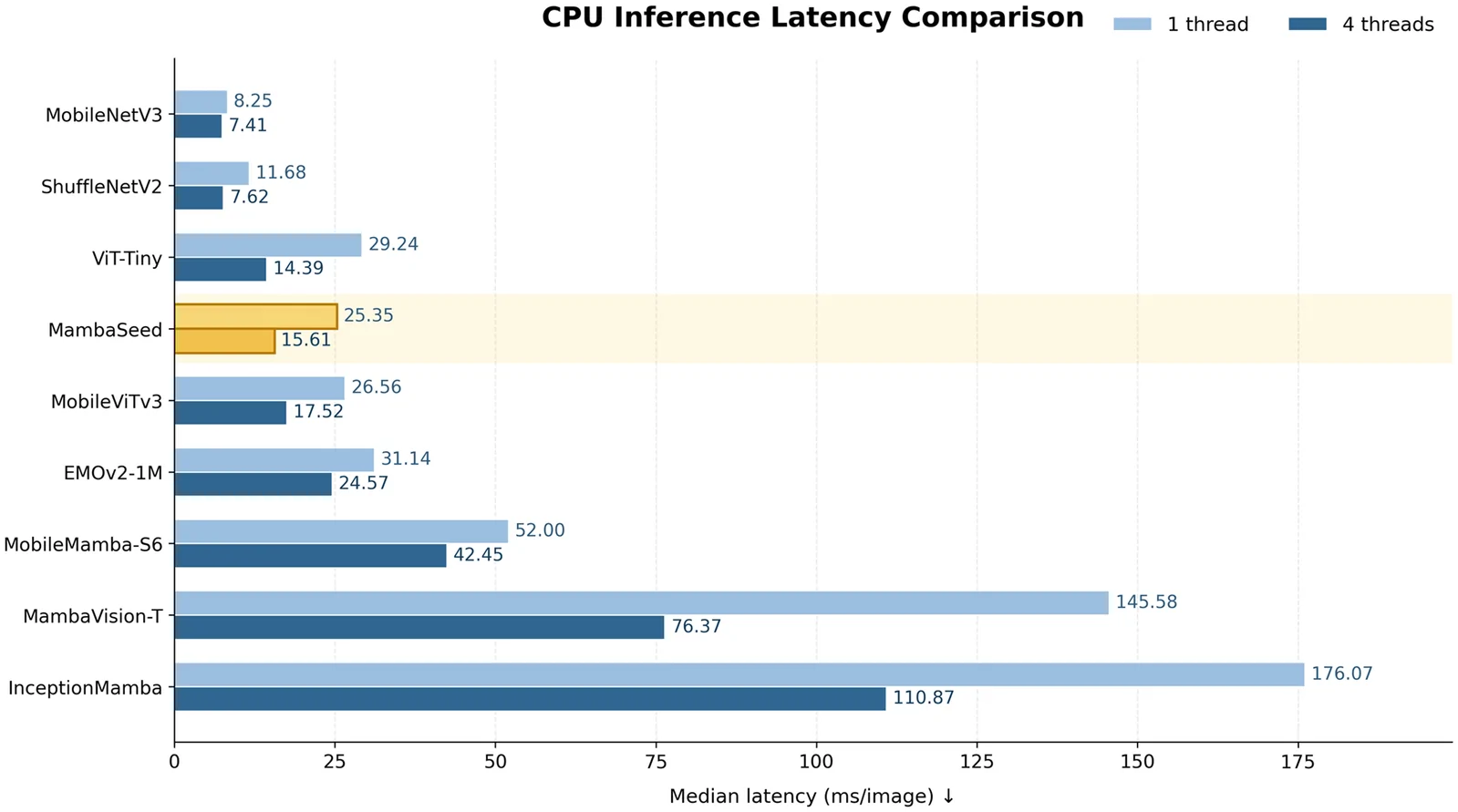
Median CPU-only forward-pass latency under one- and four-thread settings.

### Explainability analysis

Because classification scores alone do not reveal which visual evidence supports a prediction, Grad-CAM++ was used to examine the regions emphasized by MambaSeed. Fig 9 shows that the response pattern changes with symptom morphology rather than remaining fixed across classes. Compact lesions produce concentrated activation around necrotic spots and damaged tissue, whereas diseases expressed through yellowing, vein distortion, or leaf deformation elicit broader responses that follow the affected leaf structure. This distinction is particularly visible for Tomato Yellow Leaf Curl Virus and Tomato mosaic virus, whose symptoms are spatially dispersed rather than confined to a single lesion. In contrast, the healthy sample shows no compact lesion-specific hotspot and receives a more diffuse response over the leaf surface. Background regions are generally weakly activated across the diseased samples, suggesting that predictions are guided primarily by pathological textures and structural abnormalities instead of scene context. The class-dependent shift from localized to distributed attention is therefore consistent with the intended role of LCBI: preserving local detail while allowing deeper features to integrate symptom patterns across the leaf.

**Fig 9:**
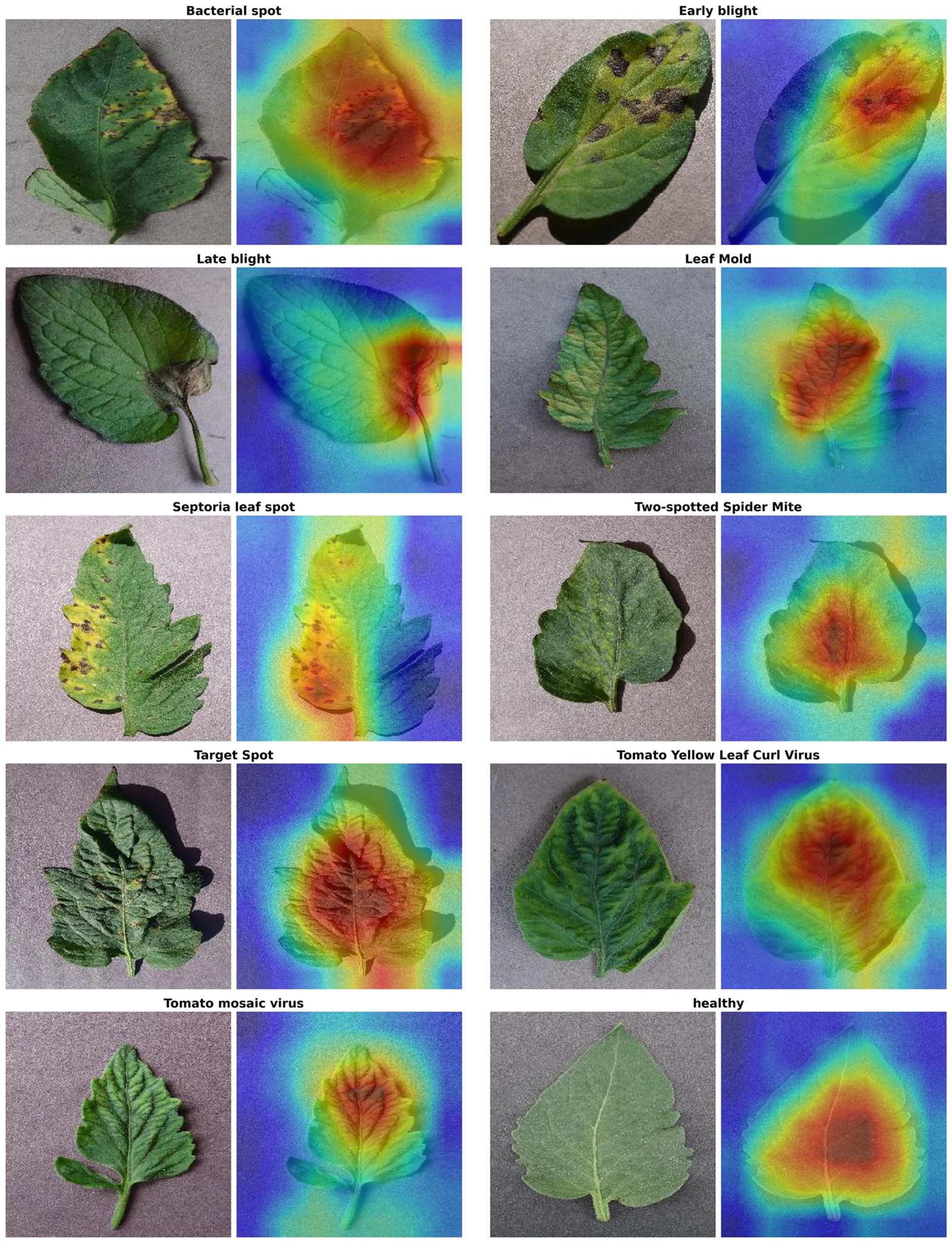
Grad-CAM++ visualization results of MambaSeed on tomato leaf disease samples.

To examine how LCBI reshapes this attention, Fig 10 follows a damaged-margin sample through the progressive ablation variants.

**Fig 10:**
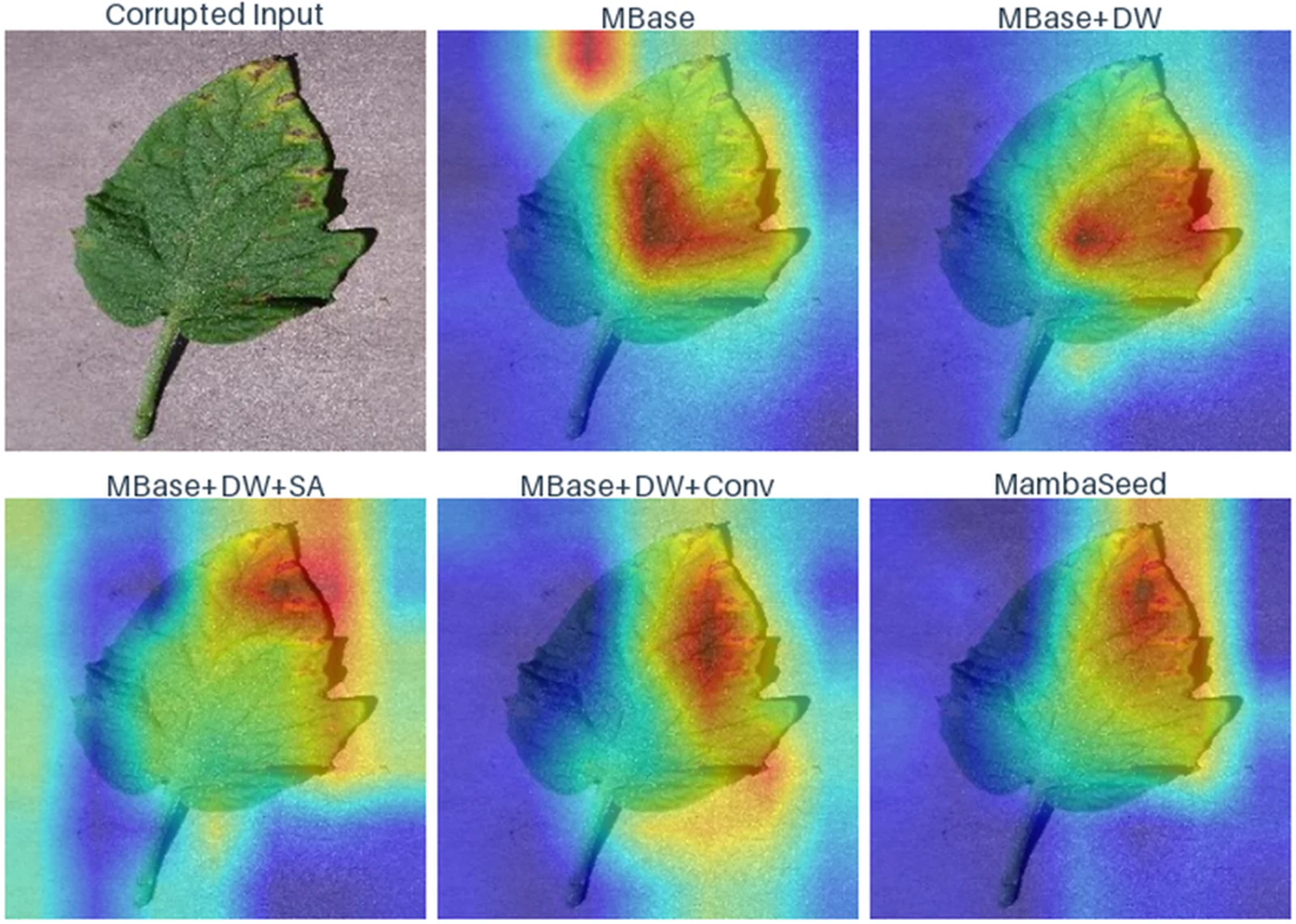
Progressive visualization comparison of LCBI ablation models.

MBase produces a scattered response that extends into the upper background, whereas adding DW shifts activation toward the right-side lesion and suppresses much of the irrelevant response. SA then broadens the response along the diseased margin, while Conv concentrates it around the damaged edge and adjacent surface, although some peripheral activity remains in both variants. With all components enabled, MambaSeed shows the most stable lesion-aligned pattern and the weakest background response. This progression complements Table 2, linking the quantitative gain to the collaboration between continuous local information injection and high-level semantic modeling rather than to any single component.

**Table 2:** Ablation results for MambaSeed.

	Stages					Expansion Factor				Components			Performance		
	2	3	4	5	6	1	2	3	4	DW	SA	Conv	Acc. (%)	F1 (%)	Loss
Version 1	✓							✓					84.15 ± 0.35	83.82 ± 0.34	0.498 ± 0.018
Version 2		✓						✓					90.95 ± 0.41	90.81 ± 0.45	0.276 ± 0.015
Version 3			✓					✓					97.18 ± 0.26	97.17 ± 0.26	0.092 ± 0.004
Version 4				✓		✓							97.98 ± 0.08	97.98 ± 0.08	0.067 ± 0.004
Version 5				✓			✓						97.95 ± 0.13	97.94 ± 0.13	0.065 ± 0.003
Version 6				✓					✓				97.80 ± 0.15	97.79 ± 0.15	0.061 ± 0.004
**MBase**				✓				✓					98.22 ± 0.03	98.21 ± 0.03	0.059 ± 0.001
Version 7					✓			✓					97.65 ± 0.22	97.65 ± 0.22	0.073 ± 0.008
Version 8				✓				✓		✓			98.32 ± 0.03	98.32 ± 0.03	0.046 ± 0.005
Version 9				✓				✓		✓	✓		98.47 ± 0.08	98.47 ± 0.07	0.047 ± 0.005
Version 10				✓				✓		✓		✓	98.50 ± 0.09	98.50 ± 0.09	0.045 ± 0.003
**MambaSeed**				✓				✓		✓	✓	✓	**98.97 ± 0.08**	**98.97 ± 0.08**	**0.037 ± 0.003**

### Ablation studies

Table 2 summarizes progressive ablations on the Tomato dataset. Starting from MBase, we varied depth and the expansion factor before adding DW, SA, and Conv individually and jointly, separating backbone choices from local compensation. Values are reported as the mean ± standard deviation across independent random initializations.

#### Ablation findings and model selection.

Table 2 traces the model design from backbone selection to the complete LCBI mechanism. Increasing MBase from two to five stages raised mean accuracy from 84.15% to 98.22%. To place this gain against the cost of further scaling, we additionally profiled MBase variants from one to seven stages, as detailed in Supplementary Table S6 in S1 Text. The six-stage model did not improve on the five-stage accuracy, despite increasing the parameter count from 1.1337M to 2.3500M and latency from 3.59 to 4.34 ms/image; a seventh stage raised these costs further to 7.3763M parameters and 5.06 ms/image. Five stages were therefore retained as a practical balance between representation capacity and lightweight inference. At this depth, *E* = 3 gave the strongest result among the tested expansion factors, while *E* = 4 added cost without improving accuracy. Building on this baseline, the local branch and the SA and Conv components progressively increased accuracy from 98.22% to 98.32%–98.50%. Their combined use in MambaSeed achieved 98.97% mean accuracy and reduced the loss from 0.059 to 0.037, showing that the full design benefits from coupling continuous local information injection with high-level semantic modeling. To further assess the statistical reliability of these ablation findings, a statistical significance analysis was performed for the four key LCBI comparisons. The performance differences between MambaSeed and MBase, MBase + DW, MBase + DW + SA, and MBase + DW+Conv all remained statistically significant after Holm correction. The adjusted *p* values were below 0.05, as reported in Supplementary Table S14 in S1 Text.

### Analysis of the high-frequency information injection capability of LCBI

To further verify the compensatory effect of LCBI on local high-frequency features, we conducted a systematic frequency-domain analysis of five ablation models: MBase, MBase + DW, MBase + DW + SA, MBase + DW+Conv, and MambaSeed. Intermediate features were analyzed by two-dimensional Fourier transform, spectrum centering, and high-frequency energy statistics. Because intermediate layers often balance local detail and global semantics [50,51], Stage 3 was selected as the primary observation point for distribution-level analysis. The threshold-sensitivity results are shown in Supplementary Fig. S2 in S1 Text.

#### Cross-stage analysis of HF ratio.

On the Tomato test set, intermediate features were extracted and analyzed through two-dimensional Fourier transform, spectrum centering, and energy statistics. HF Ratio denotes the proportion of high-frequency energy in the total spectral energy. As shown in Fig 11, MambaSeed obtained the highest HF Ratio at Stage 2 and Stage 3, reaching 0.2245±0.0529 and 0.2719±0.0486, respectively. This indicates that continuous local injection enhances high-frequency responses in key intermediate layers. At Stage 4, the highest HF Ratio appeared in MBase + DW rather than MambaSeed, suggesting that LCBI mainly alleviates intermediate-layer high-frequency attenuation instead of increasing high-frequency energy at every stage.

**Fig 11:**
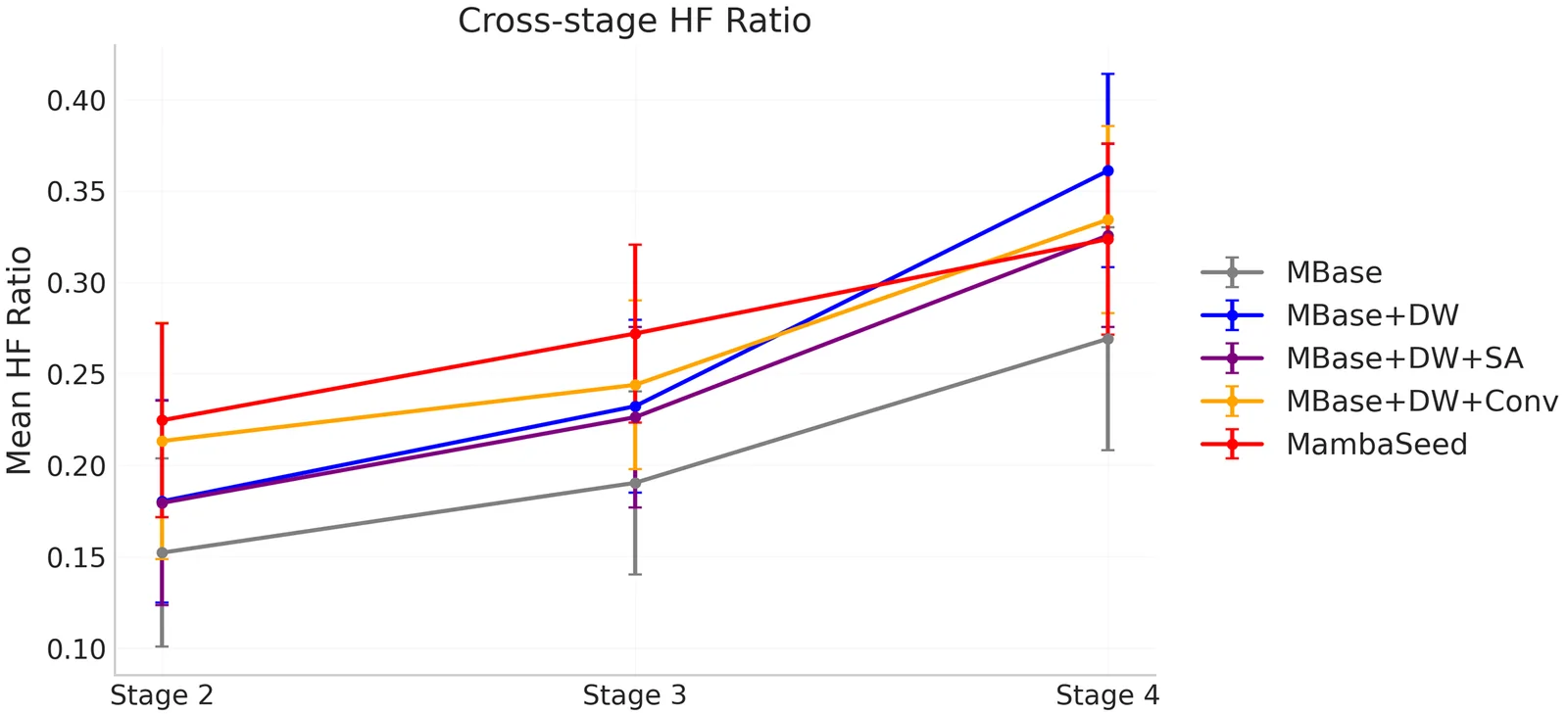
Cross-stage comparison of HF Ratio among ablation models.

#### Stage 3 distribution of high-frequency responses.

At Stage 3, HF Ratio and Top-k Energy Concentration were compared at the distribution level, as shown in Fig 12. The HF Ratio distribution of MambaSeed shifts upward, indicating stronger high-frequency retention in the intermediate layer. Its Top-k Energy Concentration is lower, suggesting that high-frequency responses are more evenly distributed rather than concentrated at a few sharp spectral peaks. In contrast, MBase shows a lower HF Ratio and a higher Top-k concentration, reflecting weaker preservation of high-frequency details.

**Fig 12:**
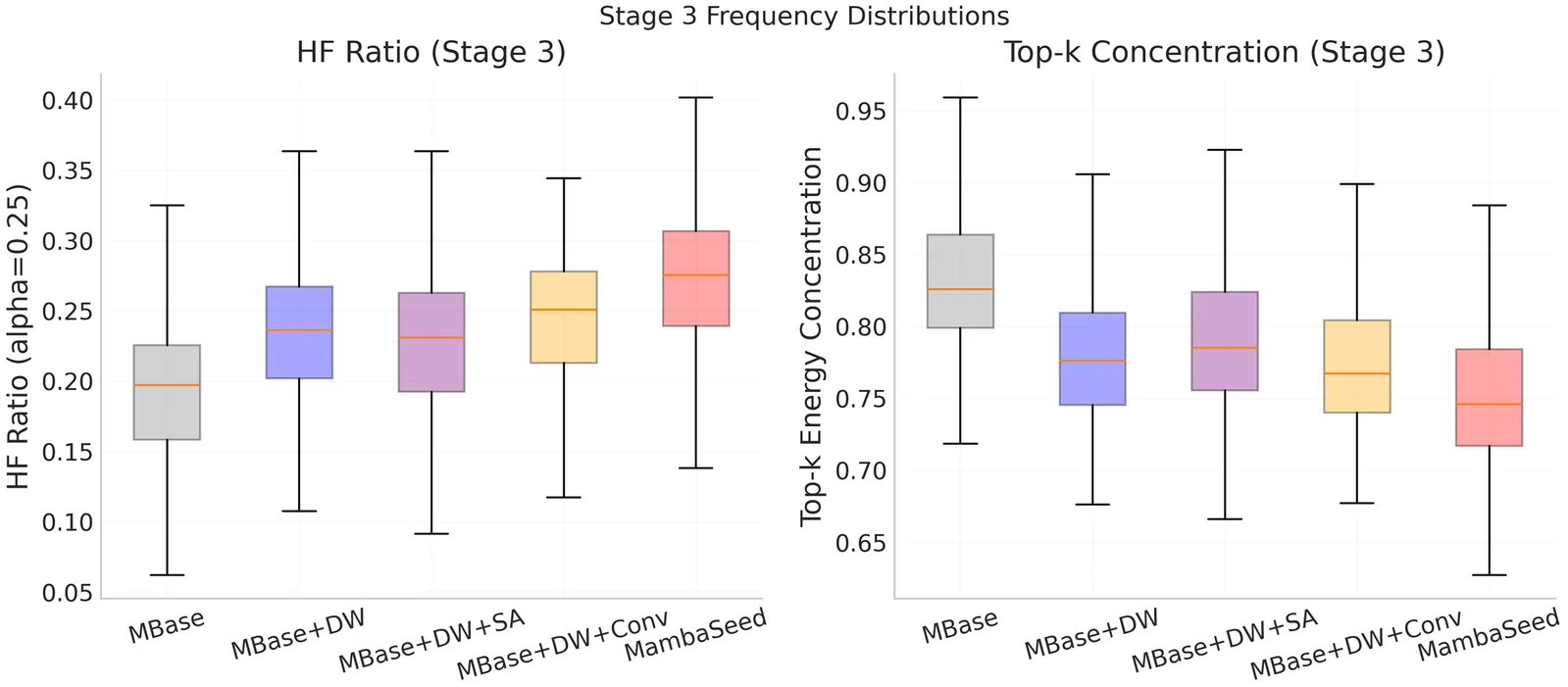
Stage-3 distribution comparison of high-frequency responses.

A qualitative visualization of the energy distribution in the high-frequency corner regions was also conducted, as shown in Fig 13. The overall response of MBase in the high-frequency corner regions is relatively weak. After the DW branch is introduced, the high-frequency regions are noticeably enhanced. MBase + DW+Conv and MambaSeed exhibit stronger and more continuous high-frequency energy distributions. This observation is consistent with the preceding HF Ratio statistics, indicating that LCBI-related designs produce stronger and more continuous high-frequency representations in intermediate layers rather than merely improving a single statistical metric.

**Fig 13:**
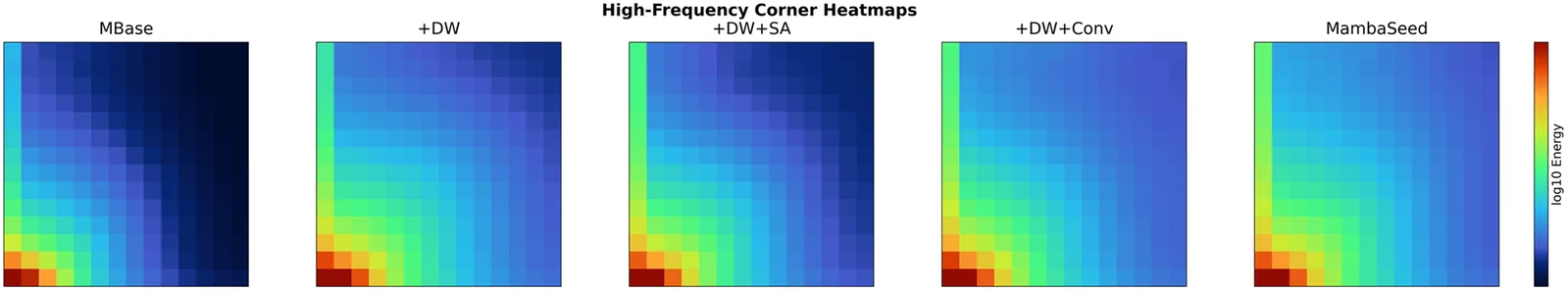
Visualization of high-frequency energy distributions in corner regions.

Taken together, the cross-stage, Stage 3 distribution, and corner-region visualization results indicate that LCBI improves intermediate-layer high-frequency retention without concentrating the response into a small number of spectral peaks. Combined with the ablation results in Table 2, this supports that continuous local information injection provides stable local texture compensation for the state-space modeling process.

#### Cross-Architecture comparison of high-frequency representations.

To place the ablation findings in a broader architectural context, we extended the frequency-domain analysis to MambaSeed and eight benchmark models, including the convolutional networks MobileNetV3 and ShuffleNetV2. Because their internal stages do not correspond directly, the comparison used the deepest 14×14 representation along each model’s execution path, followed by the same channel-wise normalization and spectral analysis. The complete feature-alignment and Fourier-analysis protocol is provided in Supplementary Table S13 in S1 Text.

As shown in Fig 14, MambaSeed produced the highest HF Ratio of 0.446 under the unified protocol, followed by InceptionMamba at 0.429, while the remaining benchmark models ranged from 0.236 to 0.360. The classical CNN MobileNetV3 reached 0.333, confirming that convolutional networks retain a meaningful high-frequency component in their intermediate representations. The stronger response observed for MambaSeed is consistent with the frequency-retention tendency observed in the LCBI ablation under a common cross-model protocol.

**Fig 14:**
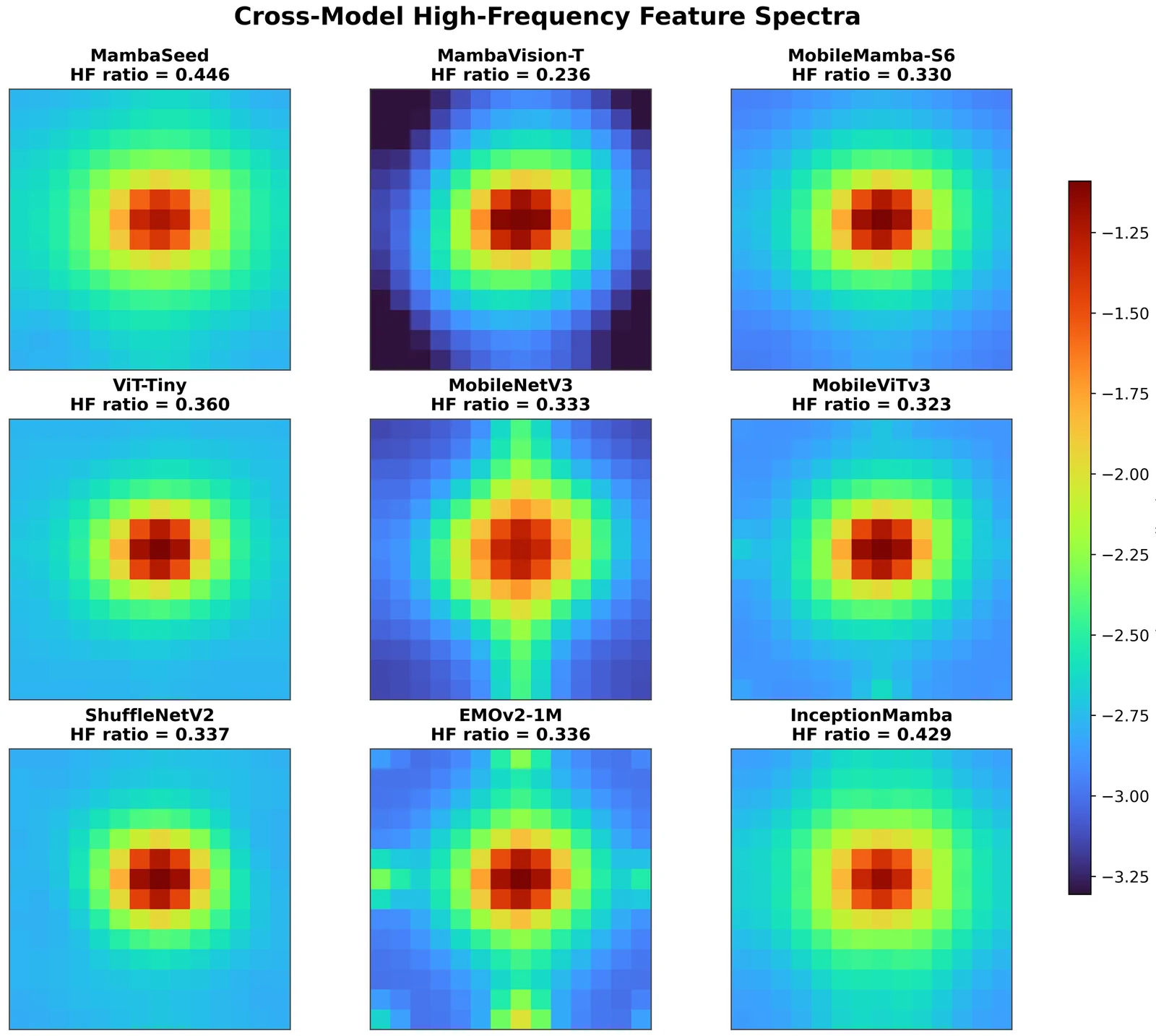
Cross-model comparison of high-frequency feature spectra under a unified representation-alignment protocol.

### Robustness analysis

To examine model behavior under controlled visual degradation, three perturbation tests were constructed: illumination variation, motion blur, and spatial occlusion. For each perturbation type, the unperturbed test images were denoted as *L*_0_, while five progressively stronger levels were denoted as *L*_1_–*L*_5_.

All robustness evaluations were conducted independently on the full test set. For each model, the three perturbation types and six severity levels produced 3×6=18 evaluation records. Overall Accuracy and Overall Macro-F1 were calculated separately as unweighted arithmetic means over these records, rather than by averaging the two metrics together:


$$\begin{array}{c} {OverallAcc}_{m}=\frac{1}{18}\sum_{c=1}^{3}\sum_{l=0}^{5}{Acc}_{m,c,l}, \end{array}$$ (10)

$$\begin{array}{c} {OverallF1}_{m}=\frac{1}{18}\sum_{c=1}^{3}\sum_{l=0}^{5}{MacroF1}_{m,c,l}, \end{array}$$ (11)

where *m*, *c*, and *l* denote the model, perturbation type, and severity level, respectively. Because each perturbation type contains the same six levels, this aggregation is equivalent to first computing the mean score for each perturbation type and then taking the unweighted mean of the three perturbation-specific scores. The clean baseline at *L*_0_ was evaluated within each perturbation family and therefore contributed three of the 18 records. The resulting overall robustness comparison is shown in Table 3.

**Table 3:** Overall robustness comparison of the evaluated models.

Rank	Model	Overall Accuracy	Overall Macro-F1
1	ViT-Tiny	0.9370	0.9378
2	MambaSeed	0.9067	0.9084
3	MambaVision	0.9039	0.9044
4	MBase + DW + SA	0.8880	0.8890
5	MBase + DW	0.8870	0.8880
6	MBase + DW+Conv	0.8855	0.8870
7	MobileMamba-S6	0.8843	0.8869
8	InceptionMamba	0.8711	0.8701
9	EMOv2-1M	0.8687	0.8698
10	MBase	0.8616	0.8612
11	MobileViTv3	0.7998	0.8011
12	MobileNetV3	0.7756	0.7750
13	ShuffleNetV2	0.6966	0.6923

Across all 18 evaluation records, ViT-Tiny ranked first, while MambaSeed ranked second with an Overall Accuracy of 0.9067 and an Overall Macro-F1 of 0.9084, narrowly ahead of MambaVision. These aggregate scores place the perturbation-specific curves in context: MambaSeed does not dominate every degradation regime, but it sustains a strong overall response while retaining the compact profile established in the preceding efficiency analysis. Figs 15–17 show how this balance changes with perturbation type and severity.

**Fig 15:**
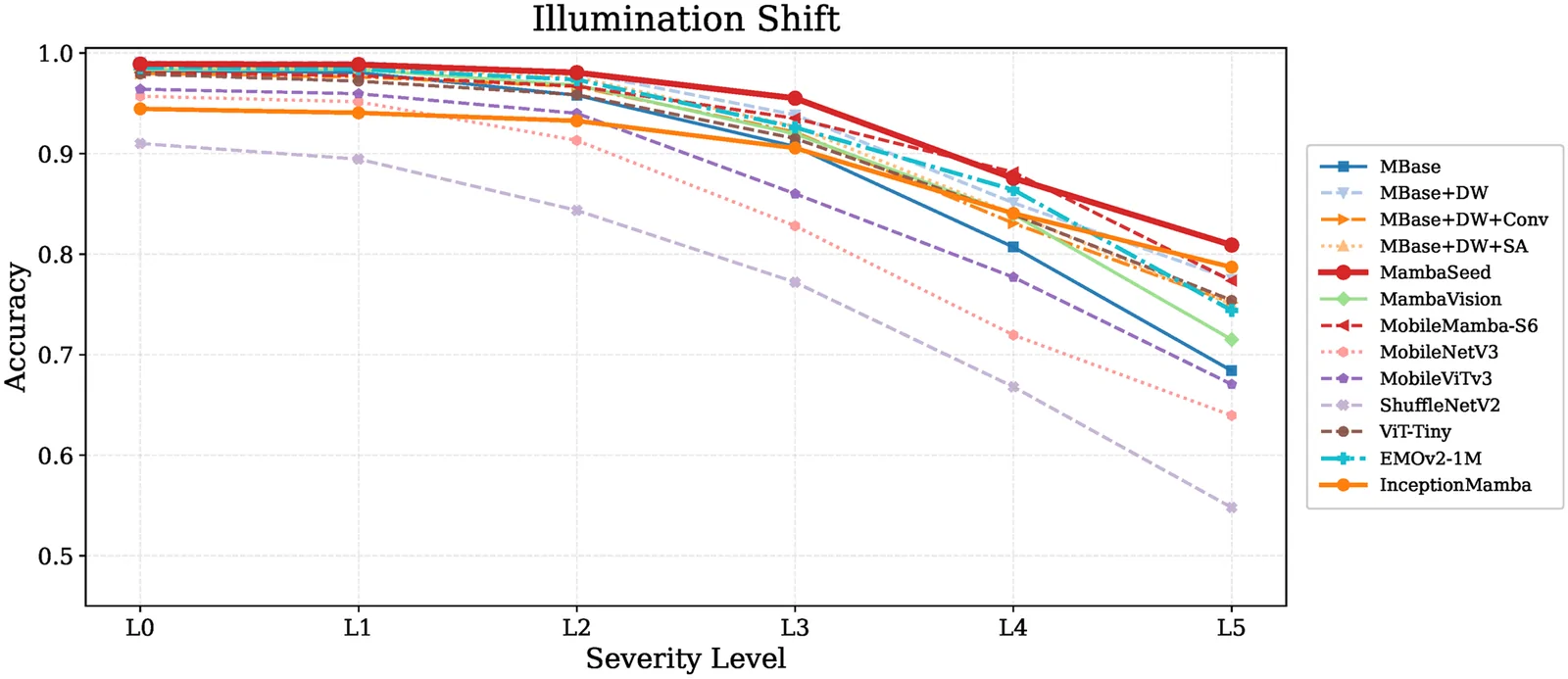
Robustness comparison under illumination variation.

**Fig 16:**
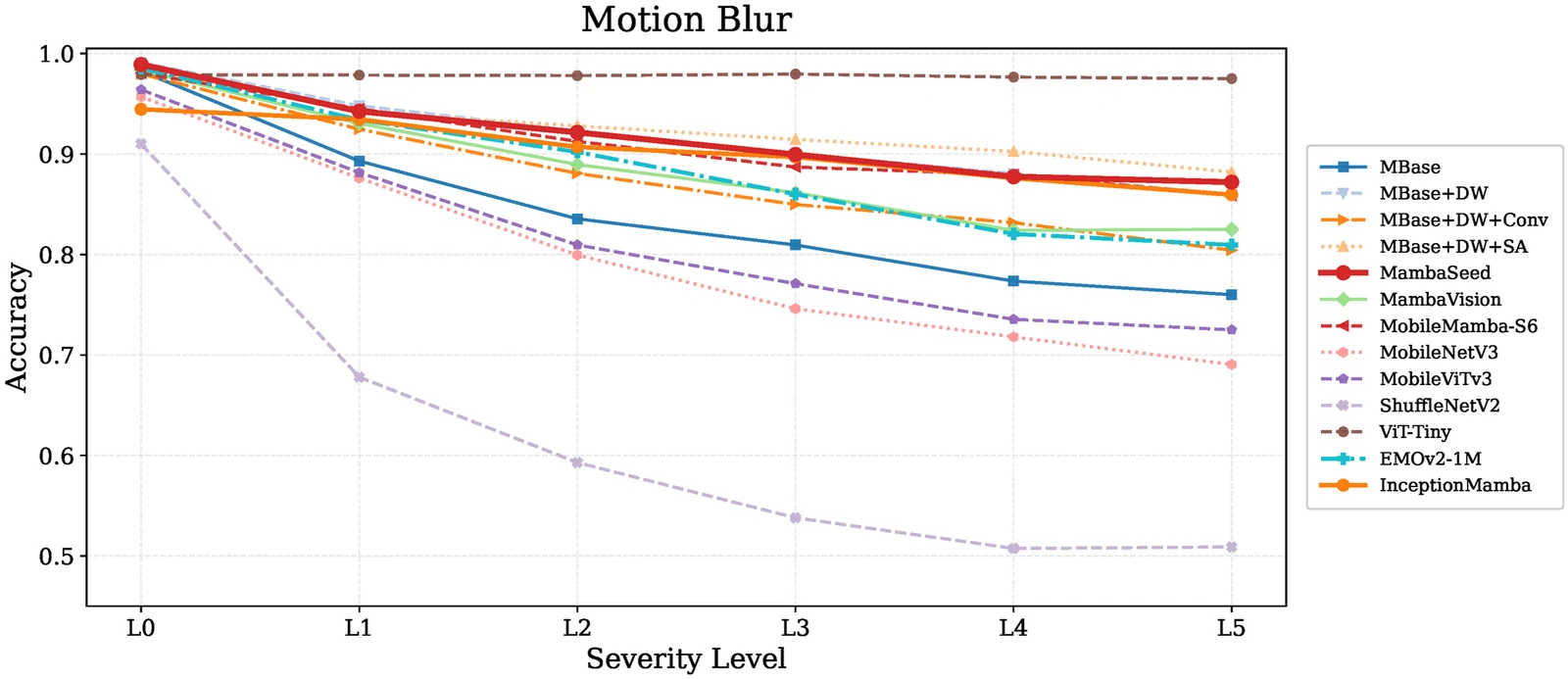
Robustness comparison under motion blur.

**Fig 17:**
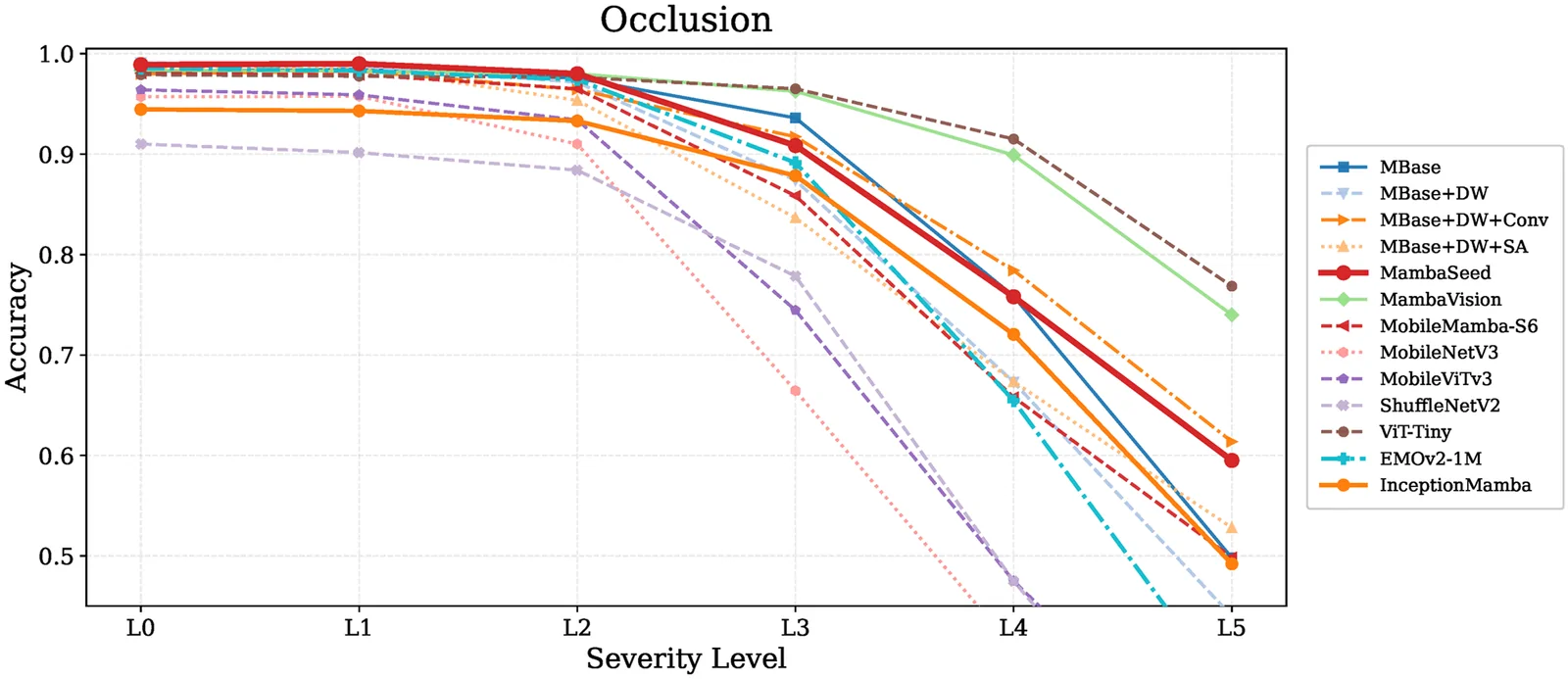
Robustness comparison under spatial occlusion.

#### Robustness to illumination variation.

Illumination variation was simulated with RandomBrightnessContrast, with brightness and contrast limits set to ±0.08×level. As shown in Fig 15, the models remain relatively close under mild changes, but their trajectories separate after *L*_2_. MambaSeed maintains the highest accuracy throughout *L*_0_–*L*_5_ and remains above both MBase and the partial LCBI variants as the shift intensifies. This pattern is consistent with the combined local and semantic pathways preserving useful lesion contrast when global brightness and contrast depart from the clean condition.

#### Robustness to motion blur.

MotionBlur and GaussianBlur were sampled with equal probability, with the kernel size increasing as k=2×level+1. Fig 16 shows a different ordering from the illumination experiment. ViT-Tiny is the most stable as blur increases, whereas MambaSeed remains in the next strongest group and consistently exceeds MBase. The partial variants further show that adding local and high-level branches moderates the loss caused by weakened lesion boundaries, although no single branch matches ViT-Tiny under the strongest blur. MambaSeed therefore retains competitive blur resistance without giving up the lightweight profile established above.

#### Robustness to spatial occlusion.

Spatial occlusion was generated with CoarseDropout using level to level+2 black blocks, each spanning 0.03×level to 0.07×level of the image height and width. The curves in Fig 17 remain tightly grouped through *L*_2_ and diverge once larger portions of the leaf are removed. ViT-Tiny and MambaVision retain the strongest accuracy under severe occlusion, while MambaSeed remains competitive through *L*_4_. Among the LCBI variants, MBase + DW+Conv slightly exceeds the final model at the two strongest levels, indicating that the complete design favors balanced behavior across perturbation types rather than specializing in extreme occlusion.

Taken together, the three tests explain MambaSeed’s second-place aggregate ranking in Table 3. ViT-Tiny provides the strongest absolute robustness, whereas MambaSeed combines competitive degradation tolerance with nearly 80% fewer parameters and 74% fewer MACs. The result is a broader balance of robustness and efficiency rather than superiority under every individual perturbation.

## Conclusion

This study presents MambaSeed, a lightweight hybrid network that uses Local Continuous Branch Injection to preserve fine-grained high-frequency cues during state-space modeling. With 1.13M parameters and 0.240G MACs, MambaSeed achieved accuracies of 98.97%, 98.06%, and 95.87% on the Tomato, Corn, and Potato datasets, respectively. Its batch-one FP32 latency was 3.39 ms/image on an NVIDIA RTX 4090, while accelerator-free CPU inference reached median latencies of 25.35 ms/image with one thread and 15.61 ms/image with four threads. Ablation and cross-architecture frequency analyses showed that LCBI strengthens intermediate-layer high-frequency retention, while MambaSeed ranked second among 13 models in the aggregate controlled-perturbation comparison. Overall, MambaSeed offers a compact balance of accuracy, efficiency, and robustness for resource-conscious plant disease recognition.

## Supporting information

S1 Text Supplementary methodological details, class distributions, depth-scaling profiles, visualization, efficiency and frequency-analysis protocols, statistical significance analysis of key ablations, Algorithm S1, confusion matrices, and threshold-sensitivity analysis. This file summarizes supplementary experimental settings, class-wise data distributions, and additional analyses used for the MambaSeed experiments.
(PDF)
