# Supplementary material for: MambaSeed: A lightweight Mamba-based hybrid vision network for tomato leaf disease recognition
Source: PLoS One. 2026 Oct 15;21(10):e0359350. doi: 10.1371/journal.pone.0359350 (PMC13671571; doi:10.1371/journal.pone.0359350)
Supplement: Supplementary file 1 [file pone.0359350.s001.pdf]

S1 Text. Supplementary methodological details, class distributions, depth-scaling profiles, visualization, efficiency and frequency-analysis protocols, statistical significance analysis of key ablations, Algorithm S1, confusion matrices, and threshold-sensitivity analysis.

Article title: MambaSeed: A Lightweight Mamba-Based Hybrid Vision Network for Tomato Leaf Disease Recognition

Authors: XueShen Wang, Heng Zhang, Yayong Chen, YunLong Li, YuJie Qin, ZhongYu Lv, XiangJuan Liu

Overview

This supporting information file provides supplementary methodological details for the MambaSeed study. It summarizes the implementation, evaluation metrics, class-wise data distributions, depth-scaling profiles, t-SNE projection protocol, model-efficiency benchmarks, frequency-domain analyses, a statistical significance analysis of key ablations, Algorithm S1, confusion-matrix comparisons, and threshold-sensitivity analysis used for the experiments described in the manuscript.

Class Distributions and Data Partitioning

Supplementary Tables S1–S3 report the class-wise sample counts of the Tomato, Corn, and Potato datasets used in the experiments. For each category, the numbers of training, validation, and test images are shown together with the total number and its proportion within the corresponding dataset.

Data partitioning was performed independently within each class. After class-wise randomization with a fixed seed of 42, 60% of the images were assigned to the training subset, while the remaining images were divided equally between the validation and test subsets. Minor one-image differences between validation and test counts in some categories are caused by integer rounding. No image was shared among the training, validation, and test subsets, and the same fixed partitions were used for all models.

Table S1. Class-wise distribution of the Tomato dataset used in the experiments.

Class	Training	Validation	Test	Total	Proportion
Bacterial spot	600	200	200	1,000	10.00%
Early blight	600	200	200	1,000	10.00%
Late blight	600	200	200	1,000	10.00%
Leaf mold	600	200	200	1,000	10.00%
Septoria leaf spot	600	200	200	1,000	10.00%
Spider mites	600	200	200	1,000	10.00%
Target spot	600	200	200	1,000	10.00%
Yellow leaf curl virus	600	200	200	1,000	10.00%
Mosaic virus	600	200	200	1,000	10.00%
Healthy	600	200	200	1,000	10.00%
**Total**	**6,000**	**2,000**	**2,000**	**10,000**	**100.00%**

The Tomato subset used in the experiments has equal class representation, with 1,000 images in each category.

Table S2. Class-wise distribution of the Corn dataset used in the experiments.

Class	Training	Validation	Test	Total	Proportion
Gray leaf spot	307	103	103	513	13.32%
Common rust	715	238	239	1,192	30.94%
Northern leaf blight	591	197	197	985	25.57%
Healthy	697	232	233	1,162	30.17%
**Total**	**2,310**	**770**	**772**	**3,852**	**100.00%**

The Corn subset contains varying numbers of images across categories. The class-wise proportional split preserved these relative frequencies in the training, validation, and test subsets.

Table S3. Class-wise distribution of the Potato dataset used in the experiments.

Class	Training	Validation	Test	Total	Proportion
Bacterial wilt	341	114	114	569	5.82%
Early blight	1,576	526	526	2,628	26.88%
Healthy	1,365	455	455	2,275	23.27%
Late blight	1,252	417	418	2,087	21.34%
Leafroll virus	316	105	106	527	5.39%
Mosaic virus	399	133	134	666	6.81%
Nematode	40	14	14	68	0.70%
Pests	366	122	123	611	6.25%
Phytophthora	208	69	70	347	3.55%
**Total**	**5,863**	**1,955**	**1,960**	**9,778**	**100.00%**

The Potato subset retains the category frequencies of the adopted dataset. Applying the same proportional split independently to each category ensured that its class composition remained consistent across the three experimental subsets.

The unequal category frequencies in the Corn and Potato subsets have two implications for interpretation. Dataset-level accuracy is influenced more strongly by categories with larger test sets, while category-specific estimates for less frequent classes are based on fewer observations and may therefore be less stable. For this reason, these subsets are used as complementary dataset-level evaluations rather than as evidence that performance is uniform across all categories. Their aggregate results support comparison under the retained benchmark composition, while the reliability and robustness of individual less represented categories should be interpreted with appropriate caution. The proportional split does not remove the original imbalance; instead, it preserves the same class composition in training, validation, and testing. Together with the use of identical fixed partitions for every model, this prevents the splitting procedure from introducing additional distribution differences and keeps the model comparisons on a consistent basis.

Training Configurations for MambaSeed and Benchmark Models

For MambaSeed, the five-stage channel progression $(16, 32, 64, 128, 192)$ increases representation capacity as spatial resolution decreases, while one block per stage and a capped final width maintain the intended lightweight profile. The state size of 16 and the internal 1D convolution parameter ($d\_conv = 4$) of the underlying MambaVisionMixer were retained from the original mixer configuration rather than independently tuned. Four attention heads divide the 192-dimensional final-stage representation evenly without introducing excessive attention overhead, and a drop-path rate of 0.15 provides moderate regularization. The expansion factor of 3 was selected from the ablation analysis reported in the manuscript.

For the cross-model comparison, the training budget and data pipeline were kept consistent across the nine architectures while preserving each model's stated lightweight variant. All models used random initialization, the same fixed dataset partitions, and validation-based checkpoint selection. The only model-dependent optimization setting was the initial learning rate, which was limited to the two values reported in Supplementary Table S4; the test set was used only for final evaluation.

Table S4. Architecture variants, initialization, and initial learning rates used in the comparison.

Model	Exact architecture variant	Initialization	Initial learning rate
MambaSeed	MambaSeed-224	Random initialization	4×10^{-4}
MambaVision-T	`mamba_vision_T`	Random initialization	3×10^{-4}
MobileMamba-S6	`MobileMamba_S6`	Random initialization	3×10^{-4}
ViT-Tiny	`vit_tiny_patch16_224`	Random initialization	3×10^{-4}
MobileNetV3	MobileNetV3-Small	Random initialization	3×10^{-4}
MobileViTv3	XX-Small-v3, `xx_small_v3`	Random initialization	3×10^{-4}
ShuffleNetV2	ShuffleNetV2-0.5×, `scale=0.5`	Random initialization	3×10^{-4}
EMOv2-1M	`EMO2_1M_k5_hybrid`	Random initialization	4×10^{-4}
InceptionMamba	VSSM; depths $(2, 2, 4, 2)$, dimensions $(96, 192, 384, 768)$	Random initialization	4×10^{-4}

Beyond these two learning-rate groups, every model followed the shared protocol in Supplementary Table S5. This matched design avoids unrestricted model-specific tuning and makes the scope of the comparison explicit.

Table S5. Shared training, preprocessing, and model-selection protocol for all nine models.

Item	Shared setting
Input resolution	224×224
Batch size and training budget	Batch size 64; 50 epochs
Loss and optimizer	Cross-entropy loss; AdamW
AdamW settings	$\beta_1 = 0.9$, $\beta_2 = 0.999$, $\epsilon = 1 \times 10^{-8}$, weight decay = 0.05
Learning-rate schedule	`CosineAnnealingLR`, $T_{\max} = 50$, $\eta_{\min} = 0$
Additional optimization	No warm-up, gradient clipping, label smoothing, Mixup, or CutMix
Training augmentation	Resize; random horizontal flip; random rotation within $\pm 15°$; color jitter with brightness, contrast, and saturation = 0.2; tensor conversion; ImageNet normalization
Validation and test processing	Resize; tensor conversion; ImageNet normalization; no random augmentation
Normalization	Mean $(0.485, 0.456, 0.406)$; standard deviation $(0.229, 0.224, 0.225)$
Data-loader workers	4
Checkpoint selection	Highest validation accuracy
Use of test set	Final evaluation only after validation-based checkpoint selection

Depth-Scaling Profile of the MBase Backbone

The accuracy ablation in Table 2 of the manuscript shows how performance changes as the MBase backbone is deepened. Supplementary Table S6 complements that comparison by tracing the accompanying growth in channel width, parameter count, computational cost, and latency from one to seven stages. In particular, extending the five-stage configuration adds progressively wider stages and increases model size and latency, providing the efficiency context for retaining five stages in the lightweight backbone.

Table S6. Complexity and latency profile of MBase variants with different numbers of stages.

Stages	Channel dimensions	Params, M	MACs, G	Latency, ms/image
1	$(16,)$	0.007562	5.759001	12.801
2	$(16, 32)$	0.029146	0.812450	1.440
3	$(16, 32, 64)$	0.111322	0.268292	2.177
4	$(16, 32, 64, 128)$	0.431706	0.237638	2.823
5	$(16, 32, 64, 128, 192)$	**1.1337**	0.240025	**3.59**
6	$(16, 32, 64, 128, 192, 256)$	**2.3500**	0.246947	**4.34**
7	$(16, 32, 64, 128, 192, 256, 512)$	**7.3763**	0.259330	**5.06**

t-SNE Projection Protocol

Supplementary Table S7 reports the complete preprocessing and projection settings used to generate the three-dimensional t-SNE visualization in the manuscript. All models were evaluated using the same Tomato test images in a fixed order, and no model-specific t-SNE tuning or projection selection was performed.

Table S7. Unified t-SNE projection protocol used for the feature-distribution visualization.

Item	Setting
Dataset and samples	Tomato test set, 2,000 images
Sample order	Fixed `ImageFolder` order, `shuffle=False`
Input size	224×224
Image normalization	ImageNet mean [0.485, 0.456, 0.406], std [0.229, 0.224, 0.225]
Feature source	Penultimate embedding before the final classifier; logits were not used
Embedding dimensions	MambaSeed 192; MambaVision-T 640; MobileMamba-S6 448; ViT-Tiny 192<br>MobileNetV3 1280; MobileViTv3 512; ShuffleNetV2 1024; EMOv2-1M 180; InceptionMamba 768
Standardization	`StandardScaler`, fitted separately for each model
PCA preprocessing	Reduced to 50 dimensions with `svd_solver="full"`
t-SNE output dimension	3
Perplexity	50
Learning rate	`auto`, with an effective value of 50
Initialization	PCA
Iterations	3,000
Random state	42
Distance metric	Euclidean
Early exaggeration	12
Optimization method	Barnes–Hut
Angle	0.5
`n_iter_without_progress`	300
`min_grad_norm`	1e-7
Software version	scikit-learn 1.7.2
Model-specific tuning	None
Projection selection	One fixed projection per model; no best-looking projection was selected

GPU-Based Model-Efficiency Protocol

The parameter, MAC, and GPU-latency results in Fig. 7 were obtained from instantiated 10-class model architectures under a common benchmark. Supplementary Tables S8–S11 document the environment, model variants, and measurement procedures used for this comparison.

Table S8. Hardware and software environment for the GPU-based efficiency benchmark.

Item	Configuration
GPU	NVIDIA GeForce RTX 4090
GPU memory	24,564 MiB
NVIDIA driver	580.105.08
Operating system	Linux 5.15.0-97-generic, x86_64, glibc 2.35
Python	3.10.13
PyTorch	2.6.0+cu124
CUDA build version	12.4
cuDNN	9.1.0
`ptflops`	0.7.5
`timm`	1.0.15
`mamba-ssm`	2.2.4
Numerical precision	FP32; automatic mixed precision was disabled

Table S9. Common configuration and instantiated variants used in the GPU-based efficiency benchmark.

Item or display name	Configuration or instantiated variant
Common output	10-class classification head
Input	Standard-normal random tensor of shape $1 \times 3 \times 224 \times 224$
Random seed	0
Model execution	`eval()` with gradient calculation disabled using inference mode
cuDNN autotuning	`cudnn.benchmark=True`
Memory format	Contiguous NCHW
Process isolation	Each model measured in a fresh subprocess
MambaSeed	Original MambaSeed-224 model configuration
MambaVision-T	`mamba_vision_T`
MobileMamba-S6	`MobileMamba_S6`
ViT-Tiny	`vit_tiny_patch16_224`
MobileNetV3	MobileNetV3-Small
MobileViTv3	`xx_small_v3`
ShuffleNetV2	Width/scale 0.5×
EMOv2-1M	Author-released `EMO2_1M_k5_hybrid`
InceptionMamba	Author-released VSSM configuration with depths [2, 2, 4, 2] and dimensions [96, 192, 384, 768]

Classification checkpoints were not required for these architecture-level measurements. Parameter count and MACs are determined by the instantiated graph and tensor shapes, while the dense GPU execution path uses the same shapes irrespective of the learned parameter values.

Table S10. Parameter-count and MAC-estimation protocols.

Item	Procedure
Parameter count	Sum of `numel()` over all tensors returned by the model parameter iterator
Reported parameter unit	Millions of parameters, $N_{\text{params}}/10^6$
Excluded from parameter count	Non-parameter buffers, including running means and variances
MAC estimation tool	`ptflops` 0.7.5
Input supplied to `ptflops`	$(3, 224, 224)$ with an assumed batch size of 1
Backend consistency	The same `ptflops` backend produced all reported values; no fallback counter was used
Reported MAC unit	10^9 multiply–accumulate operations

MACs provide a tool-dependent analytical estimate rather than a direct count of GPU instructions. Custom or functional operators not recognized by `ptflops` may be omitted, so small differences should be interpreted within this shared measurement convention.

Table S11. GPU latency protocol.

Item	Configuration
Device	Single NVIDIA GeForce RTX 4090
Input	One FP32 tensor of shape $1 \times 3 \times 224 \times 224$, created directly on the GPU
Batch size	1
Warm-up	30 forward passes
Timed iterations	100 forward passes
Timing clock	Python `time.perf_counter()`
CUDA synchronization	Immediately before and after the timed loop
Reported latency	Synchronized elapsed time divided by 100
Summary statistic	Arithmetic mean per forward pass
Included operations	Model forward execution and kernel-launch overhead within the forward calls
Excluded operations	Model construction, device transfer, input allocation and transfer, file I/O, preprocessing, and post-processing

GPU clocks, power limits, and exclusive-process mode were not modified or controlled. The reported values therefore characterize performance under the stated environment rather than a hardware-guaranteed lower bound.

Accelerator-Free CPU Inference Protocol

Supplementary Table S12 records the matched CPU-only benchmark used to compare all models without a GPU or other hardware accelerator. The protocol isolates model execution from image loading and preprocessing and applies the same timing procedure to every architecture.

Table S12. CPU-only inference-latency protocol.

Item	Configuration
Evaluated models	All compared models
Processor	Intel Xeon Gold 6430 CPU
Computing device	CPU only
Hardware accelerator	None
Architecture	x86_64
Operating system	Linux 5.15.0-97-generic, glibc 2.35
Input shape and precision	$1 \times 3 \times 224 \times 224$, FP32
Batch size	1
Intra-op threads	1 thread and 4 threads
Inter-op threads	1
Process isolation	Each model evaluated in a separate process
Warm-up	10 forward passes
Random-seed experiments	5
Timed iterations per run	20
Samples per thread setting	100
Timing function	`perf_counter_ns` around each forward pass
Summary metrics	Median latency, P90 latency, and FPS converted from the median
Latency scope	Model forward pass only
Excluded operations	Image loading, decoding, resizing, and normalization

Algorithmic Procedure of MambaSeed

The overall computational procedure of MambaSeed is provided as Algorithm S1 in algorithm format.

Algorithm S1 Algorithmic procedure of MambaSeed

Input: Image $I \in \mathbb{R}^{H \times W \times 3}$
Output: Classification logits z

$X \leftarrow \mathrm{StemBlock}(I)$
for $s = 1$ to 5 **do**
  if $s > 1$ **then**
    $X \leftarrow \mathrm{GELU}(\mathrm{BN}(\mathrm{Conv}_{3\times3}^{\mathrm{stride}=2}(X)))$
  end if
  for each block in stage s **do**
    if $s = 5$ and the block is the last block **then**
      $T_0 \leftarrow \Pi(X)$
      $R^{a}_{\mathrm{local}} \leftarrow \mathrm{GELU}(\mathrm{BN}(\mathrm{Conv}_{3\times3}(X)))$
      $T_1 \leftarrow \mathrm{RI}(T_0; \mathcal{D}_p(\mathrm{MHSA}(\mathrm{LN}(T_0))))$
      $T_2 \leftarrow \mathrm{RI}(T_1; \mathcal{D}_p(\mathrm{MLP}(\mathrm{LN}(T_1))))$
      $X \leftarrow \mathrm{RI}(\Pi^{-1}(T_2); R^{a}_{\mathrm{local}})$
    else
      $T \leftarrow \Pi(X)$
      $R^{m}_{\mathrm{global}} \leftarrow \Pi^{-1}(\mathcal{D}_p(\gamma \odot \mathcal{M}_\theta(\mathrm{LN}(T))))$
      $R^{m}_{\mathrm{local}} \leftarrow \mathrm{Conv}_{1\times1}(\mathrm{GELU}(\mathrm{BN}(\mathrm{DWConv}_{3\times3}(X))))$
      $X \leftarrow \mathrm{RI}(X; \{R^{m}_{\mathrm{global}}, R^{m}_{\mathrm{local}}\})$
    end if
  end for
end for
$f \leftarrow \mathrm{Mean}(\mathrm{LN}(\Pi(X)))$
$z \leftarrow \mathrm{Linear}(f)$
return z

Confusion-Matrix Comparisons

Figure S1 compares the class-wise confusion patterns of MambaSeed with eight baseline models. The baselines include MambaVision-T, MobileMamba-S6, ViT-Tiny, MobileNetV3, MobileViTv3, ShuffleNetV2, EMOv2-1M, and InceptionMamba. All matrices follow the same class order, with correct predictions shown along the blue main diagonal and misclassifications represented by the off-diagonal entries. The axes use the labels a–j, whose corresponding disease classes are listed in the legend. This aligned presentation allows recognition consistency and inter-class confusion to be examined directly across the nine models.

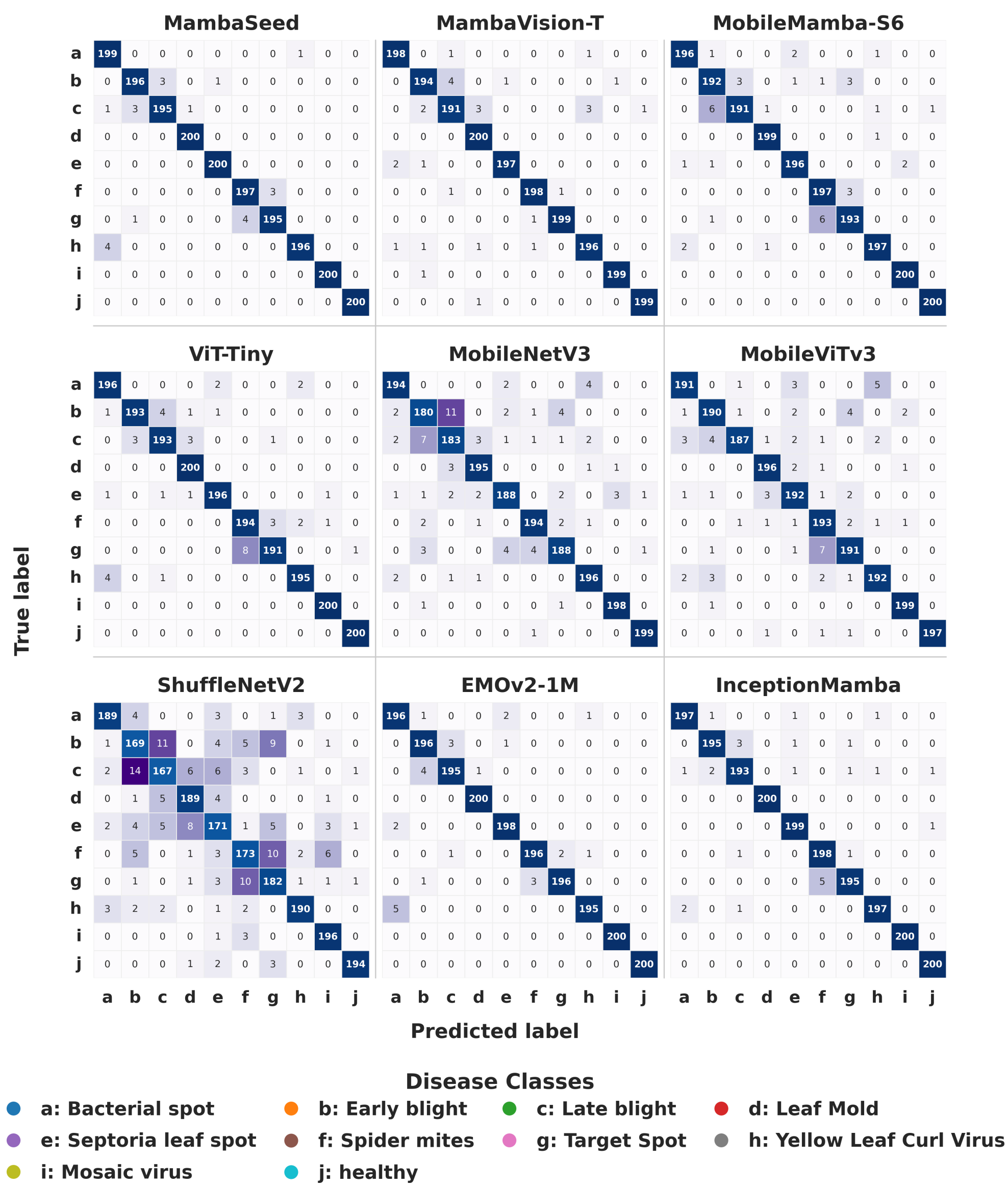

Figure S1. Confusion matrices of comparative models on the tomato leaf dataset.

Cross-Architecture Benchmark Analysis

The external comparison included MambaSeed, MambaVision-T, MobileMamba-S6, ViT-Tiny, MobileNetV3, MobileViTv3, ShuffleNetV2, EMOv2-1M, and InceptionMamba. Because these architectures do not share a strictly corresponding Stage 3, the representations were aligned using a predefined spatial criterion rather than model-specific visual selection.

Table S13. Unified protocol for cross-architecture high-frequency analysis.

Item	Procedure
Representation alignment	Deepest 14×14 representation along each model's execution path
ViT token alignment	The 196 patch tokens were reshaped to a 14×14 feature map
Spatial preprocessing	The spatial mean was removed from each channel before applying a Hann window
Spectral transform	Two-dimensional orthonormal FFT applied independently to every sample and channel
Energy normalization	Each sample and channel divided by its own total spectral energy
Aggregation	Normalized spectra averaged over all channels and 2,000 test images
Visualization scale	Common $\log_{10}$ color scale used for every model
High-frequency region	$r \geq 0.25 \times 14$
Purpose of normalization	Prevent differences in channel count and activation magnitude from dominating the comparison

Statistical Significance Analysis of Key LCBI Ablations

To assess the statistical reliability of the key ablation comparisons, five random-seed experiments were conducted for MambaSeed and four representative ablation variants. This analysis was used specifically to assess the statistical reliability of the key performance differences. Five random-seed experiments per model were summarized for this analysis, and the corresponding mean accuracies are reported together with standard deviations. Two-sided Welch's t-tests were used for the four MambaSeed-versus-ablation comparisons, with Holm correction applied for multiple comparisons; adjusted $p < 0.05$ was considered statistically significant.

Table S14. Statistical significance analysis of key LCBI ablations.

Comparison	MambaSeed mean ± SD	Comparator mean ± SD	Accuracy difference in percentage points	Raw p	Holm-adjusted p	Significant
MambaSeed vs MBase	99.00 ± 0.05	98.22 ± 0.03	+0.78	5.30×10^{-8}	$\mathbf{2.12 \times 10^{-7}}$	Yes
MambaSeed vs MBase+DW	99.00 ± 0.05	98.31 ± 0.07	+0.69	5.30×10^{-7}	$\mathbf{1.59 \times 10^{-6}}$	Yes
MambaSeed vs MBase+DW+SA	99.00 ± 0.05	98.44 ± 0.07	+0.56	6.53×10^{-7}	$\mathbf{1.59 \times 10^{-6}}$	Yes
MambaSeed vs MBase+DW+Conv	99.00 ± 0.05	98.47 ± 0.08	+0.53	3.96×10^{-6}	$\mathbf{3.96 \times 10^{-6}}$	Yes

HF Ratio Sensitivity Analysis

To test whether this conclusion depends on the definition of the high-frequency region, sensitivity analysis was performed using different high-frequency radius thresholds, as shown in Fig S2. The threshold alpha determines the radial boundary used to separate high-frequency components from the centered spectrum; therefore, a larger alpha corresponds to a stricter high-frequency region. As alpha increases, the HF Ratio of all models decreases because fewer spectral components are included in the high-frequency statistics. However, the main relative ordering remains largely stable across different threshold settings. MambaSeed

maintains the highest or near-highest HF Ratio at Stage 2 and Stage 3, whereas MBase+DW performs better at Stage 4. This indicates that the observed advantage of MambaSeed in intermediate layers is not caused by a particular threshold choice. Instead, it reflects a relatively consistent frequency-domain tendency under different definitions of the high-frequency region. This further supports that LCBI mainly enhances intermediate-layer high-frequency representation rather than uniformly increasing the high-frequency proportion at all layers.

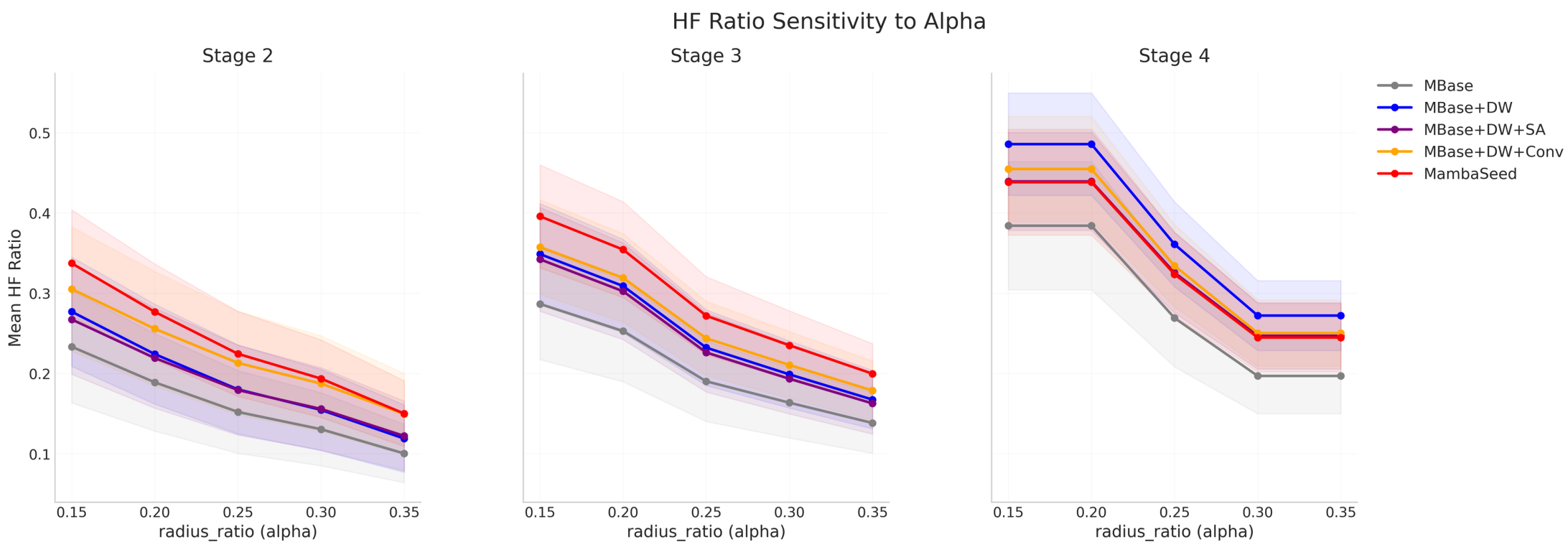

Figure S2. Sensitivity analysis of HF Ratio under different high-frequency radius thresholds.

Code Availability

The source code for the MambaSeed model is publicly available at `https://github.com/Mx2024xx/MambaSeed`.
